# Redox‐active Co(II) and Zn(II) Pincer Complexes as High‐Capacity Anode Materials for Lithium‐Ion Batteries

**DOI:** 10.1002/advs.202413656

**Published:** 2024-12-31

**Authors:** Honggyu Seong, Joon Ha Moon, Youngho Jin, Geongil Kim, Taejung Jung, Hyerin Yoo, Woonghee Lee, Kyounghoon Lee, Se Youn Cho, Jaewon Choi

**Affiliations:** ^1^ Department of Chemistry and Research Institute of Molecular Alchemy Gyeongsang National University Jinju 52828 South Korea; ^2^ Department of Chemistry University of Colorado Denver Denver CO 80204 USA; ^3^ Department of Chemical Education and Research Institute of Natural Sciences Gyeongsang National University Jinju 52828 South Korea; ^4^ Institute of Advanced Composite Materials Korea Institute of Science and Technology Wanju‐gun 55324 South Korea

**Keywords:** cobalt(II) chloride complexes, conversion reaction, high‐capacity anode materials, lithium‐ion batteries, zinc(II) chloride complexes

## Abstract

To address the ongoing demand for high‐performance energy storage devices, it is crucial to identify new electrode materials. Lithium‐ion batteries (LIBs) store energy via the electrochemical redox process, so their electrode materials should have reversible redox properties for rechargeability. On that note, redox‐active metal complexes are explored as innovative electrode materials for LIBs. Redox‐active metal(II) chloride complexes (MCC) demonstrate promising potential as anode materials, exhibiting high capacity and excellent rate capability. In particular, zinc(II) chloride complexes, referred to as ZCC, achieve a capacity of 1720 mAh g^−1^ at 2.0 A g^−1^ over 200 cycles. Additionally, the lithium‐ion storage mechanism of MCC is elucidated using ex situ analyses of MCC anode's surface in its fully discharged state. The high capacity of the MCC anode is attributed to conversion reaction with Li^+^ ions forming LiCl and electrodeposition of metallic lithium from the over‐lithiated Li^+^ ions. These findings support the potential use of redox‐active metal complexes as novel anode materials for LIBs.

## Graphical abstract

This work reports the application of redox active metal complexes as high‐capacity anode materials for lithium‐ion batteries. Cobalt and zinc chloride complex (CCC and ZCC) anodes show a high reversible capacity and rate capability. Also, ex situ analyses and density functionaltheory calculations indicate that these anode materials can store Li^+^ ions through a unique chlorine‐based conversion reaction.

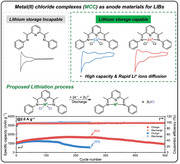

## 1 Introduction

Lithium‐ion batteries (LIBs) are widely used as efficient power sources because they are rechargeable due to the reversible electrochemical process for lithium‐ion storage. The demand for smaller devices with higher energy density has spurred the development of commercial LIBs over the past decade. However, commercial electrode materials like the LiCoO_2_ cathode (274 mAh g^−1^) and the graphite anode (LiC_6_, 372 mAh g^−1^) have major shortcomings such as low theoretical capacity, making it imperative to explore alternative candidates with higher specific capacities for the development of high‐performance LIBs. In the context of anode materials, it is recognized that metal‐based compounds can provide a significantly higher theoretical capacity than graphite via a conversion reaction, as illustrated in Equation (1).^[^ ^1^, ^2^ ^]^

(1) $$M_{x}X_{y}+\left(y\cdot z\right)Li^{+}+\left(y\cdot z\right)e^{-}\to xM^{0}+yLi_{z}X$$

Metal oxide (M_x_O_y_) as a typical metal‐based electrode material reacts with 2y equivalents of Li^+^ ion to produce x equivalents of metal atom and y equivalents of lithium oxide (Li_2_O).^[^ ^2^ ^]^ This concept is applicable to other types of compounds, including metal sulfide (M_x_S_y_), metal selenide (M_x_Se_y_), metal nitride (M_x_N_y_), and metal halide (M_x_X_y_), all of which have been extensively explored in previous studies.^[^ ^1^ ^]^

Although conversion‐type anode materials have several drawbacks, including voltage hysteresis, large volume expansion, low coulombic efficiency and poor electrical conductivity,^[^ ^1^, ^3^ ^]^ their high capacity properties still make them attractive for research. Numerous studies aimed at addressing these weaknesses have been consistently reported for over two decades.^[^ ^4^, ^5^, ^6^, ^7^, ^8^, ^9^, ^10^, ^11^ ^]^ Nevertheless, research on metal chlorides as conversion‐type anode materials has developed slowly. This is primarily due to challenges such as rapid capacity degradation caused by the shuttle phenomenon and dissolution into organic solvents. Additionally, metal chlorides are prone to degrade in the air or any chemical conditions, making their practical application difficult. Landmark research report cases of lithium storage properties in pure metal chloride anodes are CoCl_2_ and AgCl.^[^ ^12^, ^13^ ^]^ Efforts to address the dissolution issues have also led to the development of modified composite structures based on CoCl_2_, CuCl, CuCl_2_, and AgCl.^[^ ^12^, ^14^, ^15^, ^16^, ^17^, ^18^ ^]^ Most recently, Y.X. Chen et al. reported N‐doped carbon hollow cubic nanoboxes that encapsulate several particles of CoCl_2_ hydrate inside (CoCl_2_@CHCB).^[^ ^19^ ^]^ This structure demonstrated successful application potential of metal chloride with a high capacity of 920 mAh g^−1^ at 0.2 A g^−1^ over 120 cycles and cycle stability of 405 mAh g^−1^ at 2.0 A g^−1^ over 1500 cycles.

Metal complexes frequently exhibit a characteristic redox‐activity due to possible oxidation states in multiple positions of the metal center and the redox‐active ligand component.^[^ ^20^ ^]^ The redox‐activity of metal complexes can be adjusted by altering their electron structures with different ligands, even with the same metal species. Therefore, metal complexes are one of the promising research topics in the next‐generation electrode materials and are studied in various research fields that explore their redox processes.^[^ ^21^, ^22^ ^]^ Redox‐active metal complexes can be reversibly converted to a reduced or oxidized form and reverted to their initial state. If this reversible redox behavior of metal complexes could be harnessed, it would enable their application as electrode materials for lithium‐ion batteries.^[^ ^23^, ^24^, ^25^ ^]^ For example, Chen's group reported four polymeric coordination networks (polypyrrole–metal–oxygen (PPy–M–O), and polythiophene–metal–oxygen (PTh–M–O)), incorporating oxo (=O) ligands. These materials (PPy–Fe–O,^[^ ^26^ ^]^ PPy–Co–O,^[^ ^27^ ^]^ PPy–Ni–O,^[^ ^28^ ^]^ PTh–Fe–O^[^ ^29^ ^]^) revealed powerful lithium‐ion storage performances and demonstrated that lithium ion storage occurred through oxygen‐based conversion reactions, with the formation of Li_2_O, suggesting similarities to the conversion reactions of metal oxides.

**Figure 1:**
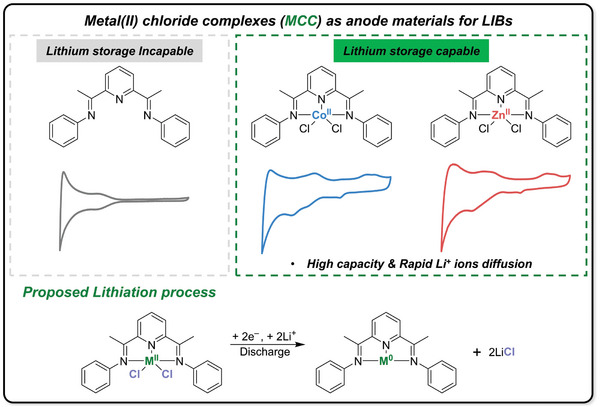
Lithium storage properties of metal(II) chloride complexes anodes.

Inspired by these previous studies, we hypothesized that if the ligand species within metal complexes could participate in conversion reaction, the metal complexes will be applied as electrode materials for LIBs. Thus, we aimed to develop a new conversion‐type anode material based on metal chlorides, which had been difficult to practical application. In this study, we synthesized two metal chloride complexes (MCC) featuring a pincer‐type ligand: a cobalt(II) chloride complex (CCC) and a zinc(II) chloride complex (ZCC). These two organometallic single molecules have excellent high‐capacity characteristics. Notably, ZCC anode delivered reversible specific capacities of 2381 mAh g^−1^ at 0.1 A g^−1^ over 100 cycles and 934 mAh g^−1^ at 2.0 A g^−1^ over 500 cycles. As shown in **Figure** **1**, the free ligand L showed no lithium storage property, whereas CCC and ZCC anodes revealed distinct electrochemical behaviors in coin‐type lithium‐half cells. This work introduces a simple yet unique concept for the lithium‐ion storage capability of MCC anodes using their chloro (Cl^−^) ligands as lithium‐acceptors through a conversion reaction mechanism.

## 2 Results and Discussion

### 2.1 Lithium‐Ion Storage Capability of MCC Anodes

**Figure 2:**
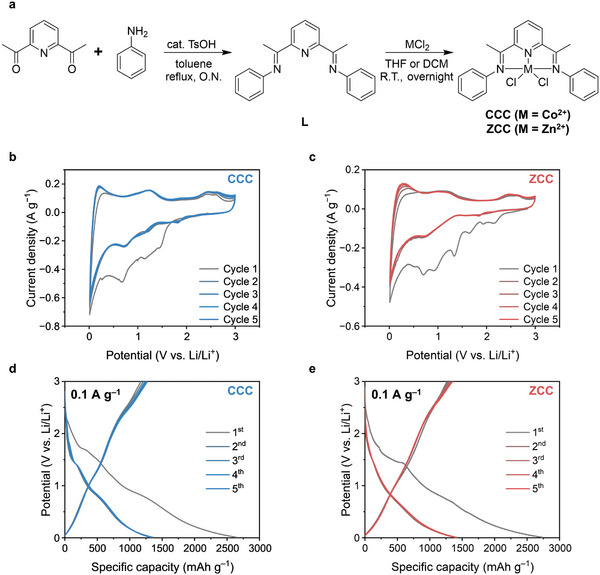
MCC synthesis and electrochemical properties of CCC and ZCC anodes in a lithium half‐cell within a potential window of 0.01–3.0 V versus Li/Li^+^. a) The synthetic scheme of pincer complex CCC and ZCC compound for the central metal of Co(II) and Zn(II), respectively. Initial 5 cyclic voltammograms of b) CCC and c) ZCC anodes at 0.1 mV s^−1^. Initial 5 galvanostatic charge and discharge profiles d) CCC and e) ZCC anodes at 0.1 A g^−1^.

**Figure** **2**a illustrates the synthetic process of 2,6‐bis[1‐(phenylimino)ethyl]pyridine MCl_2_, which was labeled as MCC. MCC represents a class of 2,6‐diiminepyridine proligand compounds. The ligand, 2,6‐bis[1‐(phenylimino)ethyl]pyridine (L), was synthesized through a condensation reaction between 2,6‐diacetylpyridine and aniline, forming the imine (C═N) bond.^[^ ^30^ ^]^ The ligand L was characterized by ^1^H nuclear magnetic resonance (NMR) spectroscopy with reference to the reported structural information (Figure S1, Supporting Information).^[^ ^30^, ^31^ ^]^ As documented in previous literature,^[^ ^31^, ^32^ ^]^ an equal molar ratio of the desired metal(II) chloride and L were stirred in an organic solvent, obtaining precipitates of CCC (cobalt(II) chloride complex) and ZCC (zinc(II) chloride complex). Finally, both were collected by filtration and after they were vacuum‐dried; CCC appeared as dark green powder, and ZCC as a pale yellow powder. The obtained CCC and ZCC powders were also measured by ^1^H NMR spectroscopy. ^1^H NMR spectrum of ZCC was confirmed by ^1^H NMR with reference to the reported chemical shifts (Figure S2, Supporting Information).^[^ ^31^ ^]^ For CCC compound, ^1^H NMR spectrum of CCC was paramagnetically broadened due to its high spin d^7^ Co(II) center.^[^ ^33^ ^]^ Meanwhile, Figure S3 (Supporting Information) shows attenuated total reflectance Fourier transform infrared (ATR FT‐IR) spectra of L, CCC, and ZCC. Characteristic peaks for C═N stretching vibration (*v*_C═N_) of L, CCC, and ZCC were observed at 1632.7, 1627.3, and 1636.2 cm^−1^. And the peak for the C─N vibration (*v*_C─N_) in pyridine ring of L, CCC, and ZCC also appeared at 1589.8, 1585.3, and 1592.2 cm^−1^, respectively.^[^ ^34^, ^35^ ^]^ The shift of these peaks for C═N and C─N bonds of MCC indicates the coordination interaction between the metal center and nitrogen of C═N bond compared to free ligand L. Finally, MCC compounds were also confirmed by high‐resolution mass spectrometry (HRMS) (Figures S4 and S5, Supporting Information). MCC, with its simple metal pincer complex structure, was anticipated to demonstrate redox activity, so we conducted various electrochemical tests to evaluate the cycle performances of the MCC anode for LIBs. The prepared MCC anode was assembled into a coin‐type lithium half‐cell (CR2032) and its performance was assessed using cyclic voltammetry (CV) and galvanostatic charge–discharge cycle tests, as shown in (Figure 2b–e). Figure 2b,c display the initial five CV curves of the CCC and ZCC anodes, respectively, within a potential range of 0.01–3.0 V versus Li/Li^+^ at a scan rate of 0.1 mV s^−1^. These anodes demonstrated reversible electrochemical features after the initial irreversible cathodic sweep, with curve shapes appearing to be markedly similar. In the first cathodic sweep, several reduction peaks emerged, attributable to the conductive reagent Super P and the ligand L moiety, leading to the formation of the solid electrolyte interphase (SEI) layer through the decomposition of electrolyte molecules. The corresponding CV curves for Super P and ligand L are presented in Figure S6 (Supporting Information). Subsequently, a reversible reduction peak at 0.710 for CCC and 0.711 V for ZCC were observed in the second cycle. We hypothesized that Co(II)Cl_2_L or Zn(II)Cl_2_L interacted with two electrons and two Li^+^ ions, transforming into Co(0)L or Zn(0)L and two LiCl as illustrated in Equation (2) (conversion reaction). During the anodic sweep, oxidation peaks at 1.216 V for CCC and 0.982 V for ZCC were observed, indicating the reconversion of Co(0)L or Zn(0)L and two LiCl back into Co(II)Cl_2_L and Zn(II)Cl_2_L, completing the reverse process of Equation (2) (de‐conversion reaction).

(2) $$M^{2+}Cl_{2}L+2Li^{+}+2\;e^{-}\to M^{0}L+2\;\mathrm{LiCl}$$

Figure 2d,e illustrates the initial five galvanostatic charge and discharge profiles for both CCC and ZCC anodes at the current density of 0.1 A g^−1^. The charge and discharge capacities (mAh g^−1^) and coulombic efficiency (CE, %) for the CCC anode are as follows (cycle number, charge and discharge capacities, CE): (1st, 1172, 2648, 44.3), (2nd, 1206, 1360, 86.1), (3rd, 1233, 1332, 90.5), (4th, 1254, 1320, 93.4), (5th, 1271, 1319, 95.1). For the ZCC anode, the corresponding values are: (1st, 1265, 2738, 46.2), (2nd, 1291, 1413, 89.5), (3rd, 1323, 1391, 92.8), (4th, 1334, 1393, 95.0), (5th, 1347, 1400, 95.3). The charge–discharge profiles confirm the reversible lithium‐storage process, like the electrochemical behaviors observed in CV curves. Furthermore, these capacity values of CCC and ZCC anodes are much higher than their theoretical capacities (CCC: 121, ZCC: 119 mAh g^−1^), which is discussed in detail in Section 2.2.

**Figure 3:**
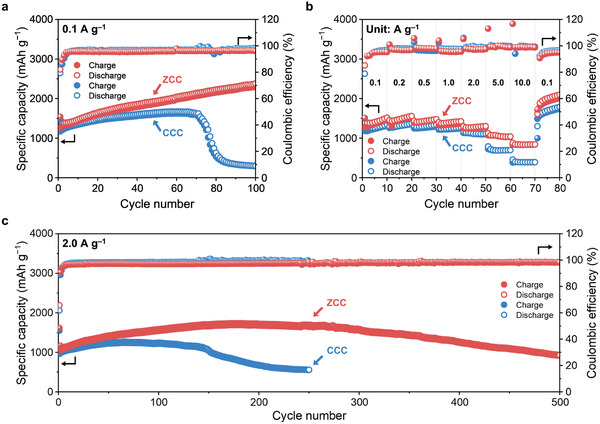
Galvanostatic constant current charge–discharge cycle performances of CCC and ZCC anodes over cycle number. Cycle performances of CCC and ZCC anodes a) at 0.1 A g^−1^ and c) at 2.0 A g^−1^. b) Rate performances of CCC and ZCC anodes at various current densities.

**Figure** **3**a illustrates the cycle performances of CCC and ZCC anodes corresponding to each cycle number at the current density of 0.1 A g^−1^. The CCC anode each showed 70th charge and discharge capacities of 1583 and 1630 mAh g^−1^ with an average CE of 95.7% over 70 cycles. After that, its specific capacities drastically were degraded and dropped to ≈300 mAh g^−1^ until 100th cycle. In contrast, the ZCC anode exhibited higher 100th charge and discharge capacities of 2301 and 2381 mAh g^−1^, respectively, compared to the CCC anode, with an average CE of 95.3% over 100 cycles. Figure 3b delineates the rate performances of CCC and ZCC anodes, demonstrating measurements of 10 charge and discharge capacities at the current densities of 0.1, 0.2, 0.5, 1.0, 2.0, 5.0, and 10.0 A g^−1^, concluding with the final 10 cycles after returning to 0.1 A g^−1^. At each 10th cycle, the performance parameters of CCC anode (current density, charge capacity, discharge capacities and CE) were as follows: (0.1, 1298, 1326, 96.7), (0.2, 1353, 1371, 97.9), (0.5, 1294, 1337, 96.2), (1.0, 1245, 1271, 97.6), (2.0, 1095, 1110, 98.6), (5.0, 696, 698, 99.8), (10.0, 391, 393, 99.3), (0.1, 1744, 1789, 96.6). Conversely, the ZCC anode demonstrated the following results: (0.1, 1470, 1518, 95.3), (0.2, 1524, 1560, 97.0), (0.5, 1459, 1481, 98.0), (1.0, 1368, 1427, 95.6), (2.0, 1274, 1304, 97.6), (5.0, 1029, 1041, 98.9), (10.0, 836, 844, 98.8), (0.1, 2010, 2099, 95.0). Overall, the rate capability of the ZCC anode surpassed that of the CCC anode. Figure 3c shows high current density performance at 2.0 A g^−1^, where the cycle performances of CCC are summarized as follows (cycle number, charge capacity, discharge capacities and CE): (1st, 957, 2063, 46.4), (2nd, 975, 1079, 88.7), (250th, 553, 559, 98.7) with an average CE of 98.4% across 250 cycles. For the ZCC, they are: (1st, 1064, 2192, 48.5), (2nd, 1064, 1169, 91.0), (200th, 1675, 1720, 97.5). ZCC anode also showed a capacity degradation after ≈200 cycles and then delivered charge and discharge capacities of 915 and 934 mAh g^−1^ at 500th cycle with an average CE of 97.5% during the cycle tests. The galvanostatic charge–discharge cycle tests concluded that the ZCC anode offers superior lithium‐ion storage performance with higher specific capacities and exceptional rate capability under rapid charging conditions compared to the CCC anode. A detailed comparison of related materials is described in Table S1 (Supporting Information), showing the remarkable cycle performance of MCC anodes.

To measure the practical full cell performances of MCC anodes, further electrochemical tests were conducted in full lithium‐ion batteries. In this work, we did not conduct any treatment to enhance their full cell performance, including pre‐lithiation and adding some additives. After optimizing the balance between MCC anodes and with NCM cathode (LiNi_0.8_Co_0.1_Mn_0.1_O_2_) as shown in Figure S7 (Supporting Information), they were fabricated into CR2032 coin‐type full cells (in Figure S8a, Supporting Information). Figure S8b (Supporting Information) shows the rate performances of CCC||NCM and ZCC||NCM full cells at different current densities of 0.1, 0.2, 0.5, 1.0, 2.0 C (1 C = 200 mA g^−1^). When it sequentially measured 5 cycles at each rate, CCC||NCM full cell delivered discharge capacities (vs NCM) of 90, 79, 67, 55, and 38 mAh g^−1^, whereas ZCC||NCM full cell 100, 91, 80, 67 and 51 mAh g^−1^. After returning to 1.0 C, CCC||NCM and ZCC||NCM full cells exhibited discharge capacities (vs NCM) of 50 and 58 mAh g^−1^, which those degraded to 38 and 45 mAh g^−1^ after further 25 cycles. An inset photo shows the ZCC||NCM full cell could turn on red LED. Figure S8c (Supporting Information) shows the cycle performances of MCC||NCM full cells at 0.1 C. The 2nd discharge capacities (vs NCM) of CCC||NCM and ZCC||NCM full cells were 97 and 109 mAh g^−1^. After 30 cycles, they were down to 66 and 77 mAh g^−1^ with a capacity retention to 2nd capacity of 68 and 71%. Figure S8d (Supporting Information) is the cell volage curves of MCC||NCM full cells corresponding to from 2nd to 10th cycles. Cell voltage curves of 5th, 10th, 15th, 20th and 25th cycles in Figure S8b (Supporting Information) were also drawn in Figure S8e (Supporting Information). Herein, the discharge capacities (vs total mass, TOT) of CCC||NCM full cell were 80, 71, 60, 49, and 34 mAh g^−1^ at 0.1, 0.2, 0.5, 1.0 and 2.0 C, whereas ZCC||NCM full cell were 89, 81, 71, 59, and 45 mAh g^−1^, respectively. Based on Figure S8e (Supporting Information), the energy densities and power densities of MCC||NCM full cells could be calculated by Equations (4) and (5) in the experimental section. Then, the Ragone plot was shown in Figure S8f (Supporting Information), in which ZCC||NCM full cell revealed more energy densities and more power densities than CCC||NCM full cell. The higher gravimetric lithium storage performance of ZCC||NCM full cell originated from the superb cycle performances of ZCC anode than CCC anodes in the lithium‐half cell.

### 2.2 Electrochemical Kinetics and Lithium Storage Mechanism of MCC Anodes

**Figure 4:**
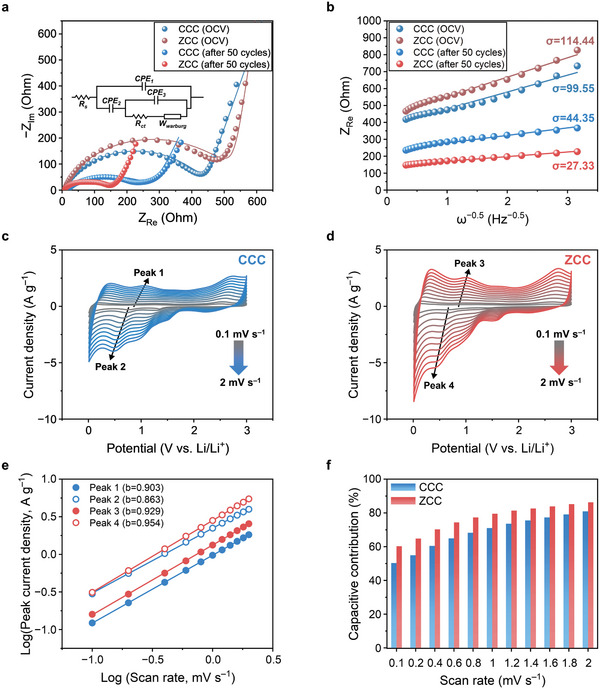
Electrochemical kinetics studies using electrochemical impedance spectroscopy and cyclic voltammetry at various scan rates. a) Nyquist plots and their fitted curves of CCC and ZCC anodes at open‐circuit voltage state and recharged state after 50 cycles at 0.1 A g^−1^ and their equivalent circuit model. b) Relationship between real impedance and the inverse of the square root of angular frequency. Cyclic voltammograms of c) CCC and d) ZCC anodes ranging from 0.1 to 2.0 mV s^−1^. e) Relationship between the logarithms of peak current density and the square root of scan rate for Peaks 1, 2, 3 and 4 in Figure 4c,d. f) Contribution of capacitive current for MCC anodes.

Electrochemical impedance spectroscopy (EIS) was conducted within the frequency range of 1 MHz to 100 mHz. **Figure** **4**a depicts the Nyquist plots and their fitted curves between −Z_Im_ and Z_Re_ within the 0 to 650 Ω range of CCC and ZCC anodes prior to the charge–discharge cycle test and after 50 cycles at 0.1 A g^−1^, and full spectrum was shown in Figure S9 (Supporting Information). In the open‐circuit voltage (OCV) state after cell assembly, the charge transfer resistances (R_ct_) for CCC and ZCC anodes were 435 and 527 Ω, respectively. These values each decreased after 50 cycles to 259 and 119 Ω. The decrease in R_ct_ for CCC and ZCC anodes suggests an enhancement in the electronic conductivity on their surfaces throughout the repeated charge–discharge cycle tests. This improvement is attributed to the interpenetration of electrolyte molecules into the electrode's inner sites.^[^ ^36^ ^]^ Figure 4b illustrates linear relationships between the real part of impedance (Z_Re_) and the inverse of the square root of angular frequency (ω) across each impedance spectrum in the low frequency region depicted in Figure 4a. The slope of each line corresponds to the Warburg coefficient (σ), which it helps determine the Li^+^ ion diffusion coefficient (D_Li_) as illustrated in **Table** **1**.^[^ ^37^ ^]^

**Table 1:** Calculated D_Li_ of CCC and ZCC anodes before and after 50 cycles at 0.1 A g^−1^.

Sample	D_Li_ [cm^2^s^−1^]	
	OCV	after 50 cycles at 0.1 A g^−1^
CCC anode	5.80 × 10^−12^	2.92 × 10^−11^
ZCC anode	4.39 × 10^−12^	7.69 × 10^−11^

As the ZCC anode exhibits a relatively higher D_Li_ value compared to the CCC anode both in the OCV state and after 50 cycles, it implies a faster transfer of Li^+^ ions on the surface of the ZCC anode than on the CCC anode. Additionally, the D_Li_ on both CCC anode and ZCC anode increased after 50 cycles, similar to the previously mentioned improvement of R_ct_. This enhanced ionic and electronic conductivity, along with the increase in specific capacity of MCC anodes during the initial cycles, is attributed to the interpenetration process of the electrolyte.^[^ ^36^ ^]^

To further explore the electrochemical properties of the MCC anode, cyclic voltammograms were recorded at various scan rates (0.1, 0.2, 0.4, 0.6, 0.8, 1.0, 1.2, 1.4, 1.6, 1.8 and 2.0 mV s^−1^). With increasing scan rates, both cathodic and anodic peaks diverged due to polarization, and their current densities increased. For peaks 1 through 4, a linear relationship was noted between the logarithms of the peak current density and the square root of the scan rate in Figure 4e. The slope (b value) of these lines indicates the electrochemical properties of each peak.^[^ ^38^, ^39^ ^]^ These b values for Peak 1, 2, 3, and 4 were 0.903, 0.863, 0.929, and 0.954 close to 1, indicating that the electrochemical reactions corresponding to these peaks undergo a capacitive‐controlled process. Total electrochemical currents of CV curves in Figure 4c,d were deconvoluted by Dunn's method.^[^ ^40^ ^]^ The contribution of capacitive‐controlled current to the total current is illustrated in Figure 4f, and capacitive contributions of CCC and ZCC anodes at 1 mV s^−1^ are detailed in Figure S10 (Supporting Information). The ZCC anode demonstrated a higher capacitive contribution than the CCC anode, indicating that the ZCC anode's surface fosters a rapid transport of Li^+^ ions, thereby enhancing rate capability better than the CCC anode.

To study the lithiation mechanism of MCC‐based anodes, pristine CCC and ZCC anodes were discharged to 0.01 V and subsequently recharged to 3.0 V, undergoing ex situ analyses by ATR FT‐IR and X‐ray photoelectron spectroscopy (XPS). For clear information on the chemical structure, a mass ratio of active materials was increased to 50 wt.%, and the electrochemical properties of 50 wt.% MCC anodes were measured, as described in Figure S11 (Supporting Information).

As shown in Figure S12 (Supporting Information), ATR FT‐IR spectra of 50 wt.% MCC anodes at different charge levels showed changes in the range from 1450 to 1750 cm^−1^. For 50 wt.% pristine CCC anode, two peaks corresponding to C═N and C─N bonds were observed at 1624.3 and 1577.5 cm^−1^, respectively. After discharging to 0.01 V, these peaks were shifted to 1628.6 and 1585.2 cm^−1^. Then, these peaks were observed at 1618.0 and 1582.3 cm^−1^ when re‐charged to 3.0 V. Likewise, those of 50 wt.% pristine ZCC anode appeared at 1636.8 and 1591.9 cm^−1^. At the discharged state to 0.01 V, they were located at 1636.3 and 1603.0 cm^−1^, and were observed at 1623.3 and 1593.4 cm^−1^ after re‐charging to 3.0 V. These results indicate that the chemical environment of C═N bond of imine group and C─N bond of pyridine coordinating with the metal centers changes during the charge–discharge process because the redox process results in the changes in chemical states of the coordinated cobalt and zinc atoms.

**Figure 5:**
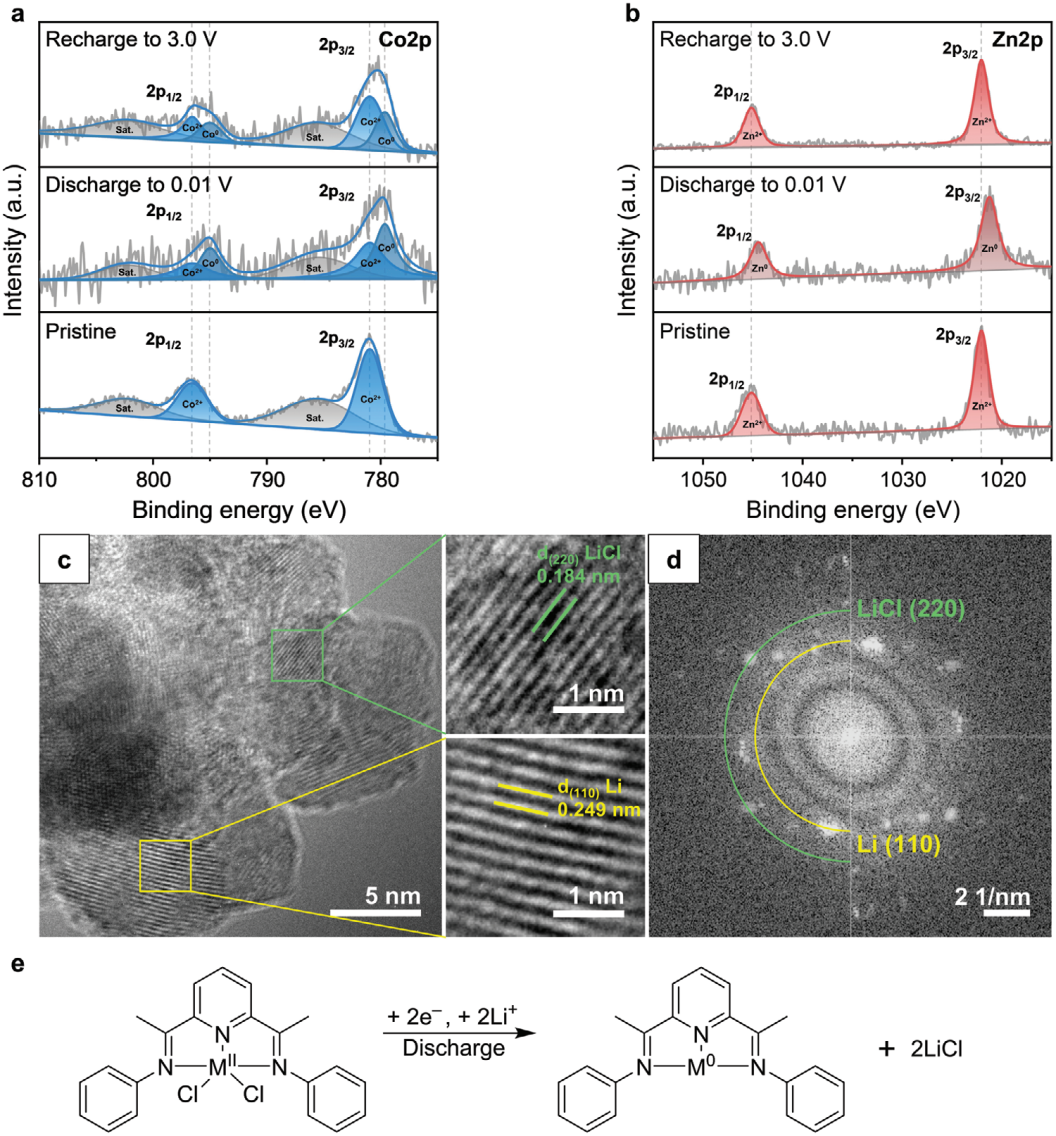
Analyses of fully discharged MCC anodes. Ex situ XPS spectra of MCC anode during the 1st charge–discharge cycle: a) Co2p region of 50 wt.% CCC anode. b) Zn2p region of 50 wt.% ZCC anode. c) HR‐TEM images of ZCC anode at a fully discharged state to 0.01 V. d) FFT patterns corresponding to Figure 5c. e) Proposed lithiation process of MCC anode.

**Figure** **5**a,b shows the ex situ XPS analysis of 50 wt.% CCC and ZCC anodes, respectively, and exhibit similar trends during the charging and discharging processes. Co2p XPS spectra of the pristine CCC anode and Zn2p XPS spectra of pristine ZCC anode indicated the presence of a metal(II) chloride complex comprising of divalent Zn(II) and cobalt(II), respectively.^[^ ^41^, ^42^ ^]^ In the Co2p regions (Figure 5a), the pristine CCC anode revealed two peaks at 796.56 (2p_1/2_) and 780.95 (2p_3/2_) eV corresponding to Co^2+^ species. When fully discharged, these two peaks for Co^2+^ species slightly diminished, and new two peaks corresponding to metallic Co^0^ species appeared at 795.00 (2p_1/2_) and 779.63 (2p_3/2_) eV. After recharging to 3.0 V, the peaks for metallic Co^0^ species were reversibly reduced, and the peaks for Co^2+^ species gathered dominant again. In the Zn2p regions for ZCC anode (Figure 5b), pristine ZCC anode showed two peaks corresponding to Zn^2+^ species at 1045.14 (2p_1/2_) and 1022.04 (2p_3/2_) eV. When fully discharged to 0.01 V for ZCC anode unlike CCC anode, only new two peaks were observed at 1044.43 (2p_1/2_) and 1021.24 (2p_3/2_) eV, indicating that the Zn^2+^ species was completely converted to metallic Zn^0^ species. Furthermore, these peaks could be restored to 1045.14 (2p_1/2_) and 1022.04 (2p_3/2_) eV corresponding to Zn^2+^ species again. The results of ex‐situ XPS analysis for the Co2p of CCC anode and Zn2p of ZCC anode indicate that their characteristic peaks at lower binding energies corresponding to the neutral chemical state of the central metal atom newly appeared and disappeared again during the charge–discharge process. Thus, their chemical state changes suggest that both Zn(II) in ZCC anode and Co(II) in CCC anode were reduced in discharged state and oxidized in a charged state. The above ex situ analysis demonstrates that the oxidation states of Co(II) and Zn(II) change during the charge–discharge process.

To acquire more chemical insights after lithiation of the MCC anode, the ZCC anode in its fully discharged state at 0.01 V underwent TEM analysis, as depicted in Figure 5c,d. The analysis highlighted the presence of (220) lattice fringes corresponding to LiCl (JCPDS No. 4–664), indicative of a conversion reaction, as illustrated in Equation (2). Additionally, (110) lattice fringes corresponding to metallic Li (JCPDS No. 15–401) were also identified.^[^ ^43^, ^44^ ^]^ These findings suggest that the high capacity of the MCC anode originates not solely from the formation of LiCl through a conversion reaction but also from the electrodeposition of nano metallic lithium on the MCC anode surface during the capacitive electrochemical process through lithiation, as illustrated in Figure 5e. Moreover, conversion‐type anode materials often exhibit extra capacities exceeding their theoretical capacities. This phenomenon is typically observed at low potentials, below ≈0.5 V, and is commonly attributed to the reversible formation and decomposition of a polymeric gel‐like SEI layer.^[^ ^45^, ^46^, ^47^, ^48^ ^]^ Similarly, the MCC anodes in this study showed capacitive storage behavior at potentials below ≈0.3 V. Thus, it is assumed that the observed extra capacity of MCC anodes is associated with the polymeric gel‐like SEI layer.

**Figure 6:**
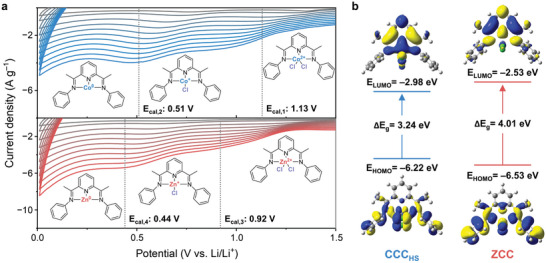
DFT‐based calculations of MCC. a) Calculated reduction potentials of CCC and ZCC anodes compared to each reductive curve of the cyclic voltammograms in Figure 4c,d. b) Frontier molecular orbital density distributions and energies of CCC_HS_ and ZCC.

When we conducted the DFT calculations at the UB3LYP/6‐311+G** level, the obtained results demonstrated reasonable lithiation pathways. Especially, CCC can exist in a low spin doublet state (CCC_LS_) or a high spin quartet state (CCC_HS_) due to its d^7^ electronic configuration, so we considered both compounds in this section. Thus, this paper adopted the approach that can achieve the approximate energies of each reduced form between two spin states based on the Poisson–Boltzmann equation.^[^ ^49^ ^]^ Detailed DFT‐calculated results of each reduced state for CCC_HS_, CCC_LS_, and ZCC are described in Figures S13–S15 and Table S2 (Supporting Information). Figures S13–S15 (Supporting Information) depict the DFT‐optimized structures considering the solvation effects for each state of CCC_HS_, CCC_LS_, and ZCC and display relative energy differences in CCC_HS_, CCC_LS_, and ZCC, when two LiCl were formed in the consecutive reduction processes of MCC anodes. And spin equilibrium constants (K_SC, n+_) of CCC at each oxidation state in Table S2 (Supporting Information) indicated that although Co(II)Cl_2_L is a well‐known high spin complex,^[^ ^33^ ^]^ Co(I)ClL and Co(0)L can be changed to low spin complexes. Based on these results, the reduction potentials of MCC anodes could be obtained by Equations (11)–(15) in the experimental section. As shown in **Figure** **6**a, the calculated reduction potentials were similar to the experimental reduction potentials of CCC and ZCC anodes, suggesting that the cathodic peaks of the cyclic voltammograms in this work correspond to the chlorine‐based conversion reaction.

Figure 6b shows the highest occupied molecular orbitals (HOMO) and the lowest unoccupied molecular orbitals (LUMO) of CCC_HS_ and ZCC anodes with band gaps (ΔE_g_) of 3.24 and 4.01 eV, respectively. Therefore, it was assumed that the R_ct_ value of CCC at OCV state was smaller than that of ZCC. Additionally, LUMOs of CCC_HS_ and ZCC were partially delocalized over the cobalt and zinc atoms as central metal species, respectively. This result indicated that cobalt and zinc atoms could sufficiently participate in the reduction process.

## 3 Conclusion

This study highlights the potential of redox‐active metal(II) chloride complexes as novel anode materials for LIBs. MCC anodes showed reversible and unique lithium storage properties in the CV measurement and galvanostatic charge–discharge cycle test, where ZCC anode, especially, delivered a high capacity of 1720 mAh g^−1^ at 2.0 A g^−1^ after 200 cycles. Ex situ analysis of MCC anodes at different charge levels by ATR FT‐IR, XPS, and TEM demonstrated that MCC anodes can store Li^+^ ions in a lithium‐half cell configuration, with their Cl^−^ ions dissociating from the central metal and reacting with Li^+^ ions to form LiCl. Moreover, not only did the capacitive electrochemical process of the MCC anode facilitate the rapid diffusion of Li^+^ ions and concentrate them on the surface of the MCC anode, but it also made the nano metallic lithium electrodeposited onto the MCC anode, revealing extra capacities beyond their theoretical capacities. Our findings highlight the potential for exploring and applying redox‐active metal complexes as next‐generation anode materials with superior cycling performance, and it is expected that numerous organometallic materials will receive increasing attention.

## 4 Experimental Section

### Materials

Chemicals used in this work were purchased from Merck and Samchun Chemical Co., Ltd. Lithium metal foil was purchased from MTI Corp. Conductive carbon black (Super P), polypropylene (PP) membrane (Celgard 3501), LiNi_0.8_Co_0.1_Mn_0.1_ (NCM811), 18 µm of copper foil, 20 µm of aluminum foil and CR2032 coin‐type cell assembly package were purchased from Wellcos Co. All purchased chemicals were used without further purification.

### Characterization

Attenuated total reflection Fourier transform infrared (ATR FT‐IR) absorption spectroscopy was conducted using a Thermo scientific US/IS5 FT‐IR spectrometer. ^1^H nuclear magnetic resonance (NMR) spectroscopy was conducted using a Bruker DRX300 300 MHz FT‐NMR spectrometer in deuterated chloroform (CDCl_3_) with chemical shift (δ) given in parts per million (ppm). Multiplicities were denoted as s (singlet), d (doublet), t (triplet), dd (doublet of doublets) and m (multiplet). High‐resolution mass spectrum (HRMS) was obtained by a Waters Xevo G2‐XS mass spectrometer (QToF MS system using electrospray ionization (ESI)). X‐ray photoelectron spectroscopy (XPS) was performed on a Thermo VG Scientific Sigma Probe spectrometer (monochromatic Al Ka radiation, hν = 1486.6 eV). Transmission electron microscopy (TEM) measurements were performed on a FEI TECNAI TF30ST at an acceleration voltage of 300 kV. All electrochemical measurements were made in a potential window of 0.01–3.0 V versus Li/Li^+^ for lithium half cells, 1.0–4.3 V for lithium‐ion full cells. EIS was conducted in a frequency range of 1 MHz to 100 mHz, using a WonAtech ZIVE SP1. CV and Galvanostatic charge–discharge cycle testing was performed on a WonAtech WBCS3000S.

### Preparation of Ligand L

First, a 250 mL one‐neck Schlenk flask equipped with a condenser was dried under vacuum.^[^ ^30^ ^]^ After the glassware was cooled to room temperature (RT), 36 g of molecular sieves, 1.6970 g of 2,6‐diacetyl pyridine (1.00 eq., 10.4 mmol) and 180 mL of toluene were added under argon flow. In this work, water dissolved in solvent was easily removed by molecular sieves, to synthesize the ligand L. After dissolving 2,6‐diacetyl pyridine, 3 mL aniline (3.16 eq., 32.9 mmol) and 100 mg p‐toluenesulfonic acid monohydrate were added under argon flow. And the mixture was stirred for 48 h at 90 °C. After the removal of molecular sieves by filtration, the solvent was removed under reduced pressure. The as‐obtained solid mixture was dissolved into a few DCM and then poured into 400 mL of saturated NaHCO_3_ aqueous solution. It was then extracted with DCM and dried over anhydrous Na_2_SO_4_. Finally, the solvent was removed under reduced pressure, and the crude was recrystallized from a mixture of THF and n‐hexane to afford bright yellow powder. Yield 2.1376 g (66%). ^1^H NMR (300 MHz, CDCl_3_) δ 8.35 (d, J = 7.9 Hz, 2H, pyridine ring), 7.88 (t, J = 7.8 Hz, 1H, pyridine ring), 7.39 (dd, J = 8.3, 7.5 Hz, 4H, phenyl), 7.16–7.09 (m, 2H, phenyl), 6.86 (dd, J = 8.4, 1.2 Hz, 4H, phenyl), 2.41 (s, 6H, ─CH_3_). ATR FT‐IR cm^−1^ 3250.2 3086.9 3055.8 3028.7 2922.1 1632.7 1589.8 1573.7 1479.8 1446.6 1422.5 1361.5 1319.0 1293.1 1246.8 1215.2 1171.7 1149.4 1111.0 1092.6 1076.6 1022.5 991.9 967.3 910.6 871.0 837.2 821.4 806.7 773.0 761.1 742.9 706.0 694.7 669.3 656.5 644.8 617.6 575.8 551.7 519.1

### Preparation of MCC

MCC powder was obtained by co‐precipitation between desired metal(II) chloride and the equal equivalent of ligand L.^[^ ^31^, ^32^ ^]^ Reaction mixture was stirred in 100 mL of the one‐neck round‐bottom flask at RT overnight (For CCC synthesis, 1.11 mmol of CoCl_2_ and L in 30 mL THF; For ZCC synthesis, 0.87 mmol of ZnCl_2_ and L in 80 mL DCM). Then, precipitation was filtrated and dried under a vacuum.

### CCC

Yield 0.2943 g (60%). ATR FT‐IR cm^−1^ 3525.3 3084.4 3035.4 2954.9 2914.2 1731.8 1627.3 1585.3 1485.5 1447.4 1370.1 1324.3 1260.0 1227.2 1187.4 1169.3 1150.0 1103.3 1069.2 1026.6 999.8 982.9 912.9 905.9 861.1 836.5 815.2 778.3 759.0 743.4 722.7 694.6 669.1 657.5 617.7 582.8 574.7 555.9 535.8. HRMS (ESI^+^ ToF, MeOH) calcd. for C_21_H_19_N_3_CoCl^+^ [M─Cl]^+^ m/z = 407.0600, found m/z = 407.0640. Related CCDC references: 690831,^[^ ^50^ ^]^ 701490 (DCM solvate).^[^ ^32^ ^]^

### ZCC

Yield 0.3847 g (98%). 1H NMR (300 MHz, CDCl_3_) δ 8.44–8.37 (m, 1H, pyridine ring), 8.19 (d, J = 7.7 Hz, 2H, pyridine ring), 5.30 (s, 4H, DCM), 2.50 (s, 6H, ─CH_3_). ATR FT‐IR cm^−1^ 3502.2 3078.0 3034.4 2959.3 2915.5 1737.6 1636.2 1592.2 1485.1 1447.5 1369.9 1321.0 1257.8 1226.7 1187.9 1169.8 1150.3 1104.4 1069.2 1024.0 1000.3 982.6 906.4 860.3 833.1 815.7 777.5 756.8 744.1 735.5 722.7 694.1 670.5 654.0 619.0 581.9 572.8 555.4 537.2. HRMS (ESI^+^ ToF, MeOH) calcd. for C_21_H_19_N_3_ZnCl^+^ [M─Cl]^+^ m/z = 412.0559, found m/z = 412.0619. Related CCDC references: 1113699 (DCM solvate),^[^ ^31^ ^]^ 1185458 (MeCN solvate).^[^ ^51^ ^]^

### CR2032 Coin‐Type Half‐Cell Fabrication

For the preparation of the MCC anode, 30 mg of MCC powder as the active material, 60 mg of conductive reagent (Super P), and 10 mg of PVDF binder were ground onto agate mortar with NMP solvent. When it became homogeneous black ink, it was bar‐coated onto 18 µm of copper foil. And then dried at 80 °C under air for an hour and then under vacuum for 3 h. The as‐prepared MCC anodes were punched into a 10 mm disc as the working electrode. Lithium foil, Celgard 3501 PP membrane, and 1 M LiPF_6_ in a 1:1 (volume ratio) mixture of ethylene carbonate (EC) and diethyl carbonate (DEC) was used as a counter/reference electrode, a separator and an electrolyte. All CR2032 coin‐type lithium half‐cells were assembled in a glove box filled with pure argon gas. (O_2_ and H_2_O < 0.1 ppm)

### CR2032 Coin‐Type Full Cell Fabrication

The preparation process of NCM cathode is similar to that of MCC anode. For the NCM cathode preparation, 80 mg of NCM811 powder as the active material, 10 mg of conductive reagent (Super P), and 10 mg of PVDF binder were used and cast on 20 µm of aluminum foil. After dried, it was punched into a 10 mm disc. Then, NCM cathode and MCC anode, Celgard 3501 PP membrane as a separator, and 1 M LiPF_6_ in a 1:1 = EC:DEC as an electrolyte were fabricated into full cell configuration. All CR2032 coin‐type lithium‐ion full cells were assembled in a glove box filled with pure argon gas. (O_2_ and H_2_O < 0.1 ppm)

### Electrochemical Analysis

The theoretical capacity of MCC anode was derived from the Equation (3).^[^ ^52^ ^]^

(3) $$Q_{theoretical}=\frac{nF}{3.6M_{w}}$$

The terms Q_theoretical_, n, F and M_w_ correspond to the theoretical capacity (mAh g^−1^), the number of electrons, the Faraday constant (9.6485 × 10^4^ C mol^−1^), and the molecular weight (g mol^−1^), respectively. In this work, n is 2 with reference to the number of Cl^−^ ions available for the chlorine‐based conversion reaction of MCC anodes.

The energy density (E) and Power density (P) were obtained by Equations (4) and (5).^[^ ^53^, ^54^ ^]^

(4) $$E=\int_{0}^{t}I\times Vdt$$

(5) $$P=\frac{E}{t}$$

where t, I and V are the step time in discharge process, discharge current density and discharge voltage.

Following Equations (6)–(10) were used to investigate the electrochemical kinetics of MCC anodes.

(6) $$Z_{Re}=R_{s}+R_{ct}+\sigma \omega^{-0.5}$$

where R_s_ represents the solution resistance.

(7) $$D_{Li}=\frac{R^{2}T^{2}}{2A^{2}n^{2}F^{4}C^{2}\sigma^{2}}$$

where R is the gas constant, T denotes the absolute temperature, A represents the electrode area, n refers to the number of transferred electrons, F indicates the Faraday constant, and C is the concentration of the Li^+^ ions in the electrolyte.

(8) $$i=av^{b}$$

(9) logi=loga+blogv

where i denotes the peak current density and v represents the scan rate, b value near 0.5 suggests that the electrochemical reaction is dominated by diffusion control. Conversely, a b value close to 1 indicates a predominantly capacitive‐controlled reaction.^[^ ^39^ ^]^

(10) $$i_{t}=k_{1}v+k_{2}v^{0.5}$$

where i_t_ is the total current density, *k*_1_ *v* represents the capacitive‐controlled current, and *k*_2_ *v* ^0.5^ represents the diffusion‐controlled current.

### Density Functional Theory (DFT) Based Calculation

All DFT‐based calculations were conducted at the UB3LYP/6‐311+G(d,p) level using the Gaussian 16W. Solvation‐free energies were calculated by using the universal solvation model based on solute electron density (SMD) with a static dielectric constant ε_EPS_ of 89.78 and an optical dielectric constant ε_EPSINF_ of 2.01 to simulate the solvation effect of ethylene carbonate electrolytes.^[^ ^55^ ^]^

The redox potentials (E^red^) versus the potential of Li/Li^+^ reference electrode of MCC anodes at the solvation state by the electrolyte condition could be derived from the Nernst Equation (Equation 11), considering the thermodynamic cycle from the gas phase in the previous articles.^[^ ^56^, ^57^, ^58^ ^]^

(11) $$\Delta E^{red}=-\frac{\Delta G_{soln}}{nF}-1.44$$

ΔG_soln_ is the difference of the Gibbs free energy using the SMD solvation model.

Furthermore, Equations (12)–(15) were used to correct the energies by calculating the relative number of CCC in both the low spin (LS) state and high spin (HS) state.^[^ ^49^, ^59^ ^]^

(12) $$\begin{array}{c} \Delta E^{red}=\Delta E_{LS}^{red}+\left(\frac{RT}{F}\right)\;\ln\left[\frac{x_{LS,n+}}{x_{LS,\left(n-1\right)+}}\right]=\Delta E_{HS}^{red}+\left(\frac{RT}{F}\right)\ln\left[\frac{x_{HS,n+}}{x_{HS,\left(n-1\right)+}}\right] \end{array}$$

(13) $$x_{LS,n+}={\left(1+K_{SC,n+}\right)}^{-1}$$

(14) $$x_{\mathrm{HS},n+}=K_{\mathrm{SC},n+}{\left(1+K_{\mathrm{SC},n+}\right)}^{-1}$$

(15) $$K_{SC,n+}=exp\left(-\frac{\Delta G_{soln,SC,n+}}{RT}\right)$$

Herein, *x*_LS,n+_/*x*_LS,(n−1)+_ and *x*_HS,n+_/*x*_HS,(n−1)+_ are the mole fractions of each spin state in a given oxidation state. K_SC,n+_ and ΔG_soln,SC,n+_ are the spin equilibrium constant and the differences (HS–LS) of the Gibbs free energy using the SMD solvation model between in the given oxidation states.

## Conflict of Interest

The authors declare no conflict of interest.

## Supporting information



Supporting Information

## Data Availability

The data that support the findings of this study are available in the supplementary material of this article.
