# Supplementary material for: Redox‐active Co(II) and Zn(II) Pincer Complexes as High‐Capacity Anode Materials for Lithium‐Ion Batteries
Source: Adv Sci (Weinh). 2024 Dec 31;12(9):2413656. doi: 10.1002/advs.202413656 (PMC11884583; doi:10.1002/advs.202413656)
Supplement: Supplementary file 1 — Supporting Information [file ADVS-12-2413656-s001.docx]

Supporting Information

Redox-active Co(II) and Zn(II) Pincer Complexes as High-capacity Anode Materials for Lithium-ion Batteries

Honggyu Seong, Joon Ha Moon, Youngho Jin, Geongil Kim, Taejung Jung, Hyerin Yoo, Woonghee Lee, Kyounghoon Lee, Se Youn Cho* and Jaewon Choi*


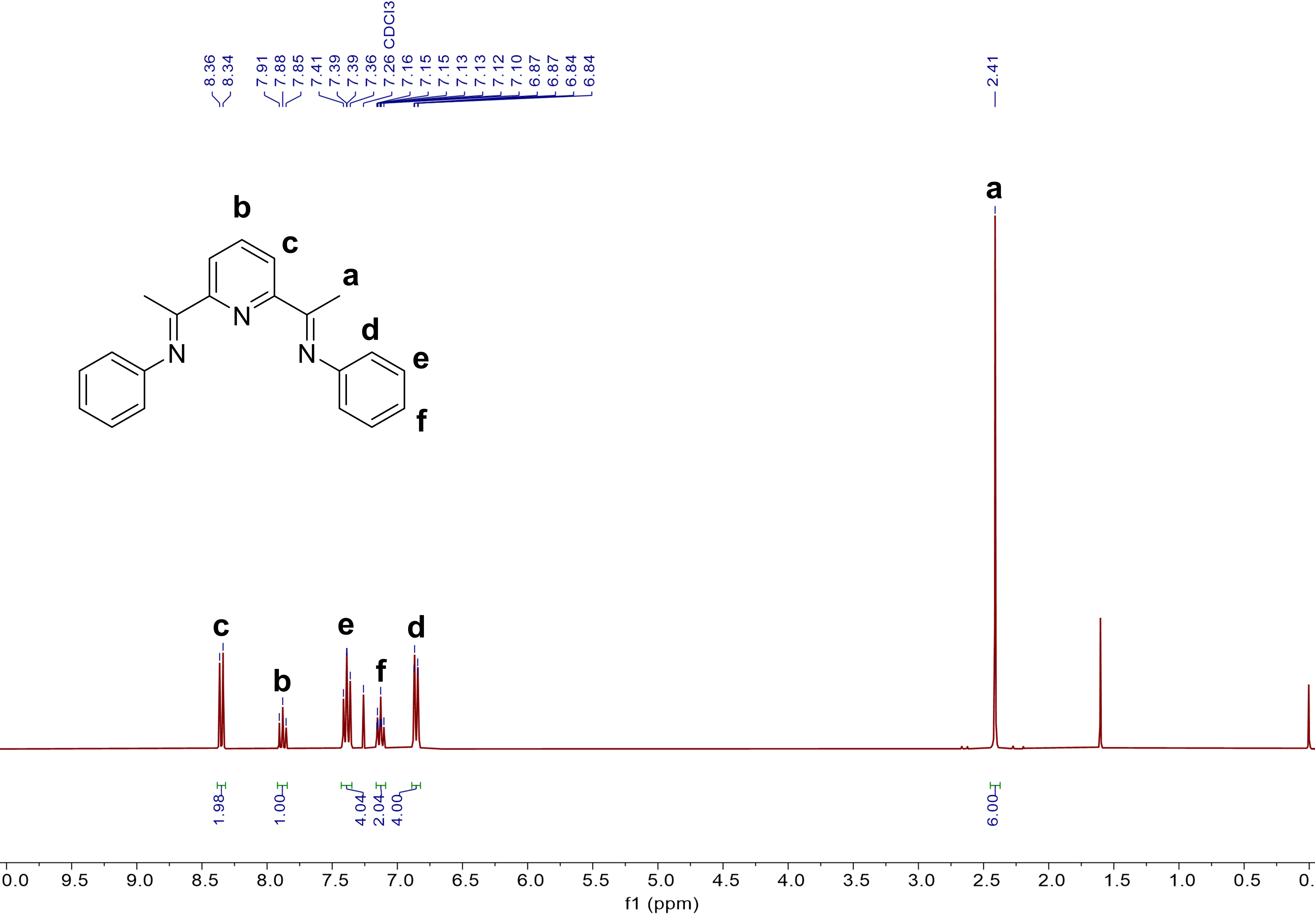


**Figure S1.** ^1^H NMR spectrum (300 MHz, CDCl_3_) of ligand L.


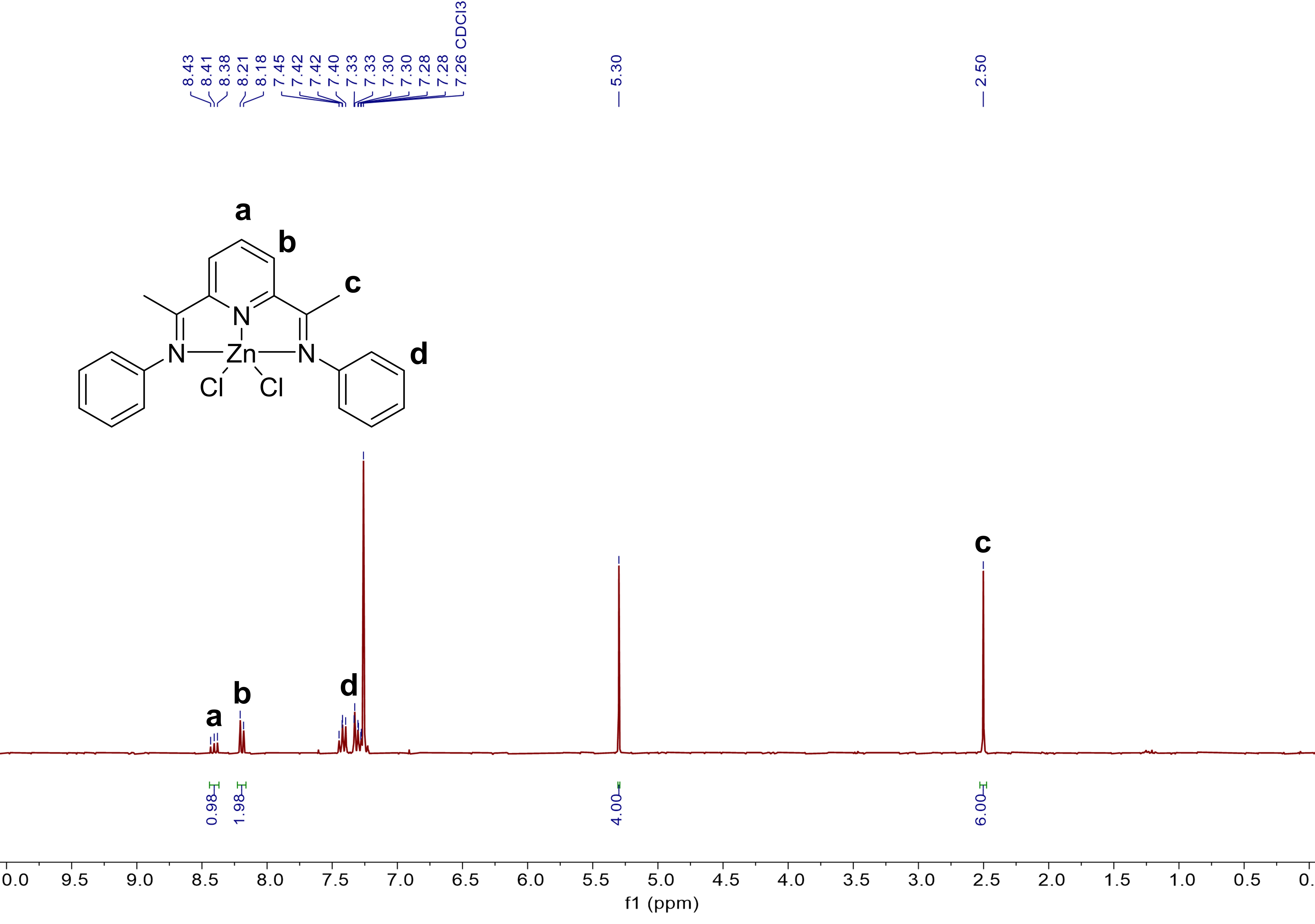


**Figure S2.** ^1^H NMR spectrum (300 MHz, CDCl_3_) of ZCC.


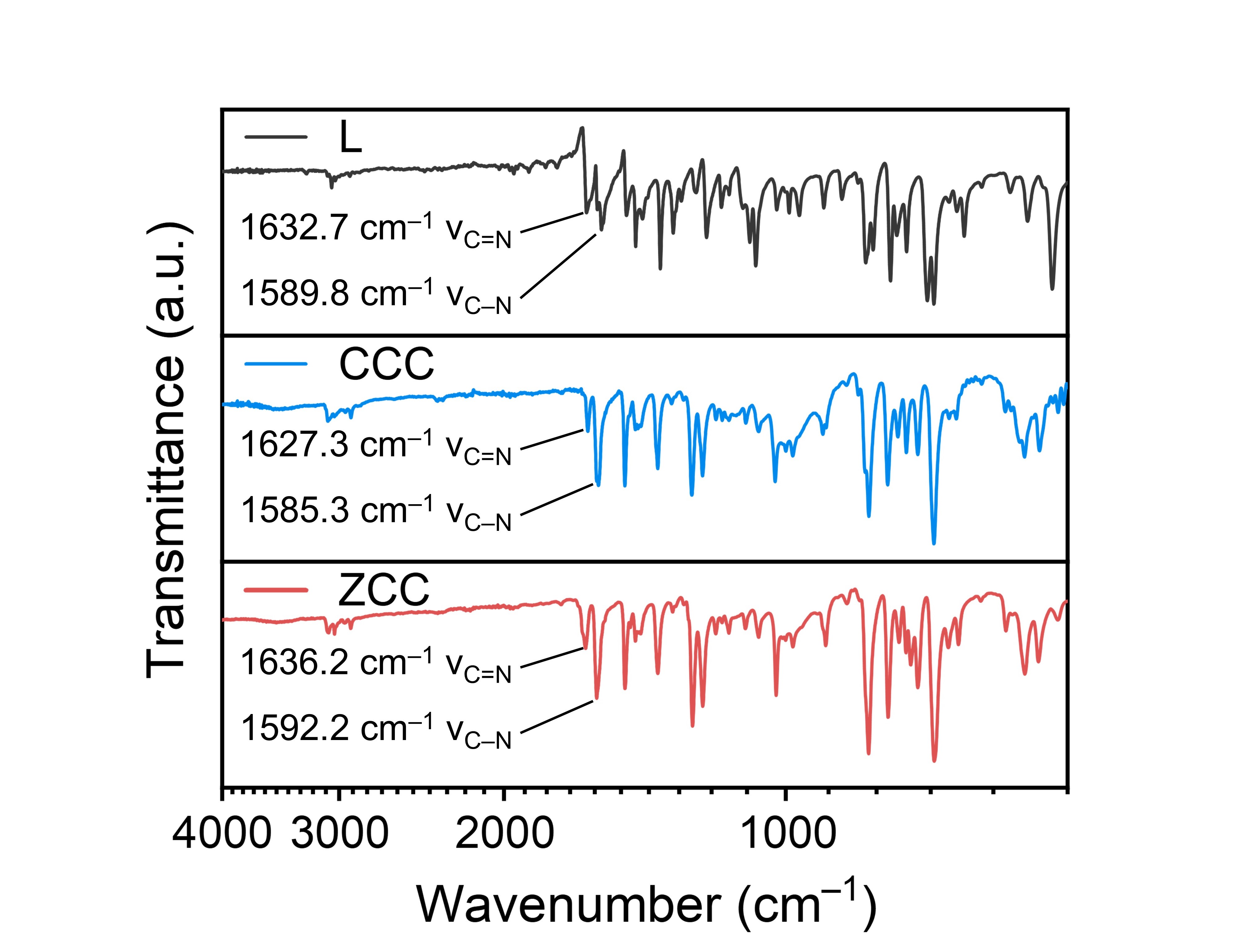


**Figure S3.** ATR FT-IR absorption spectra of L, CCC and ZCC powder.


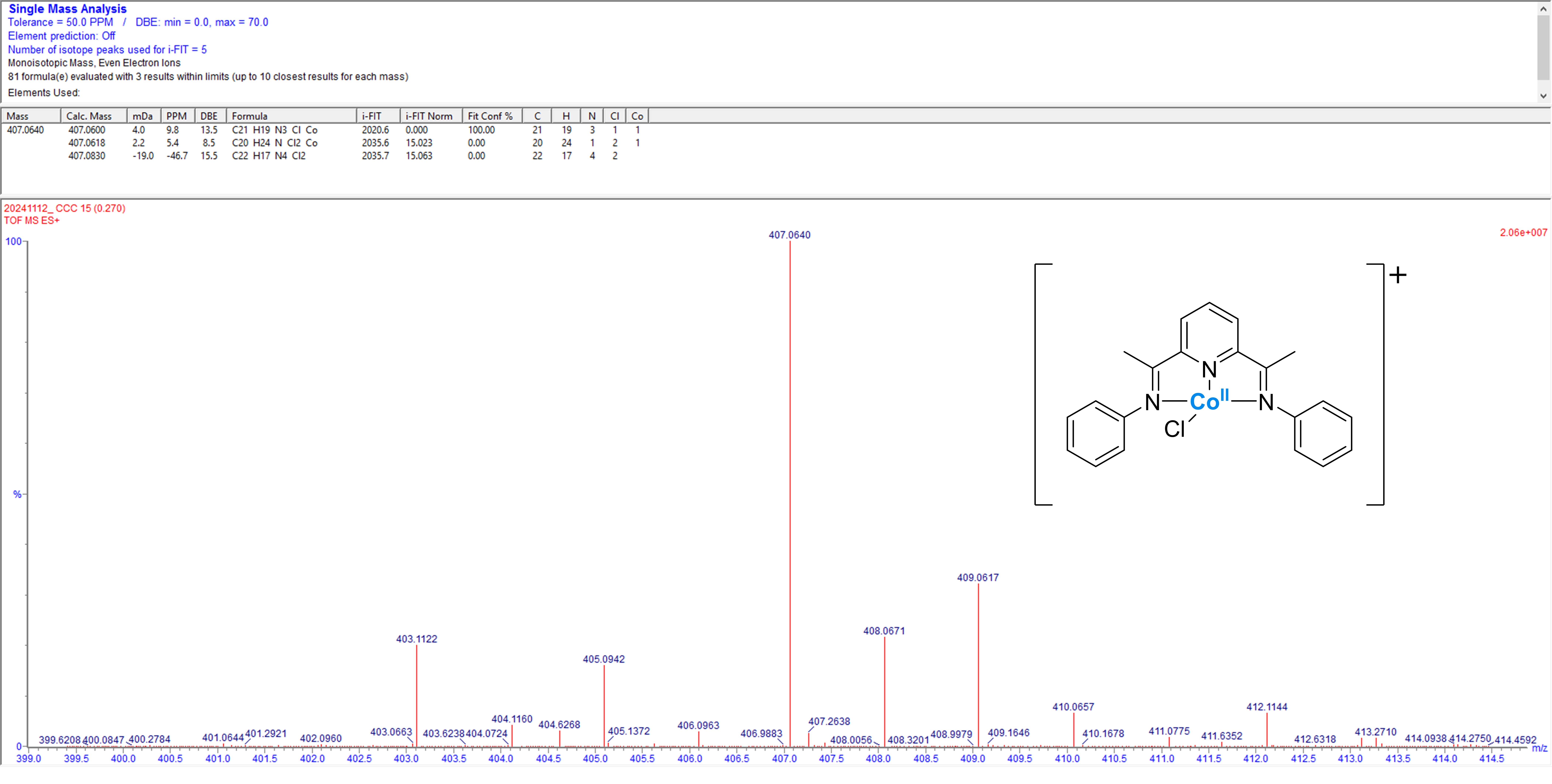


**Figure S4.** High-resolution mass spectrum of CCC.


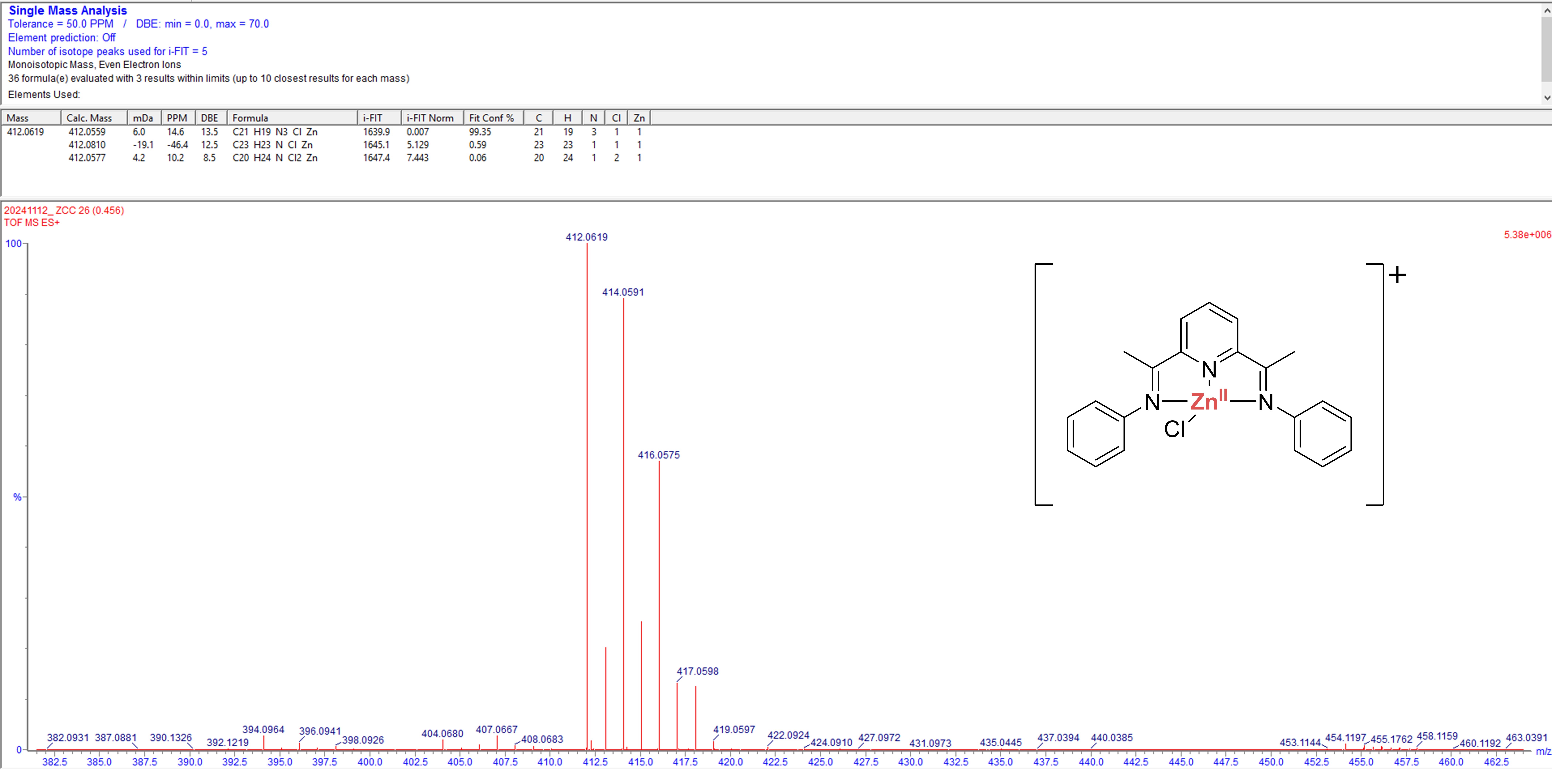


**Figure S5.** High-resolution mass spectrum of ZCC.


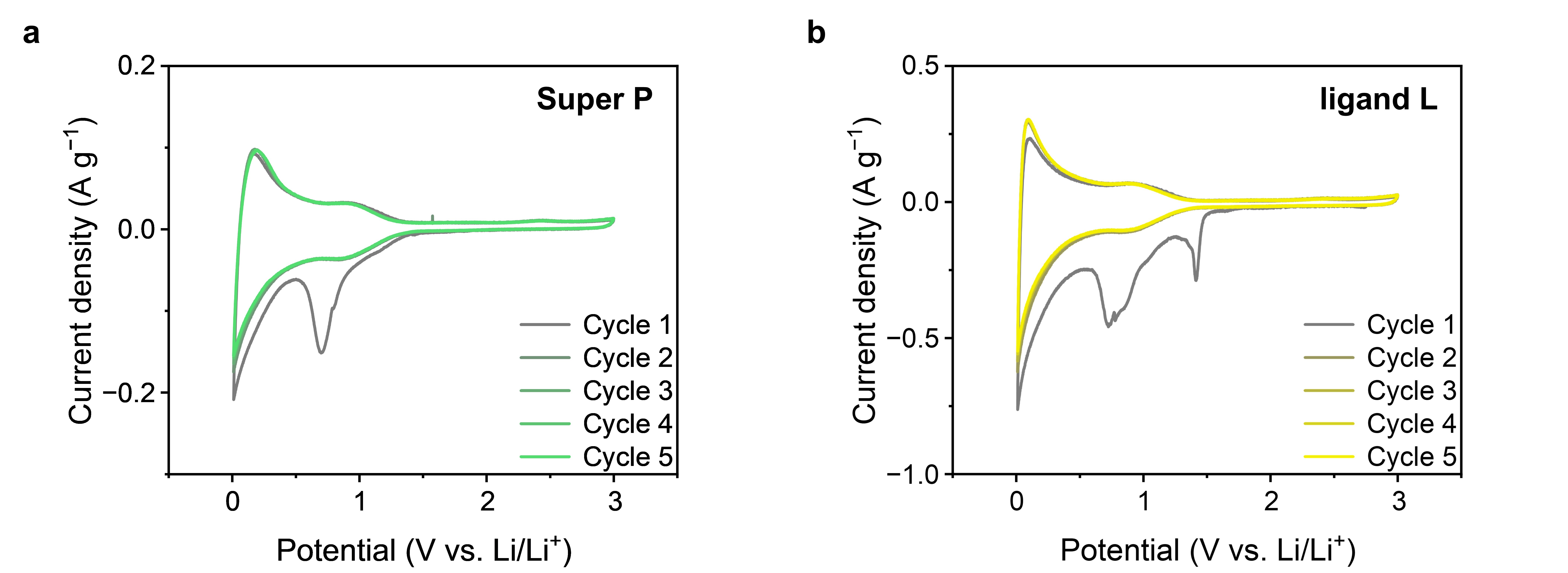


**Figure S6.** Initial 5 cyclic voltammograms of a) Super P and b) ligand L anodes at 0.1 mV s^–1^.


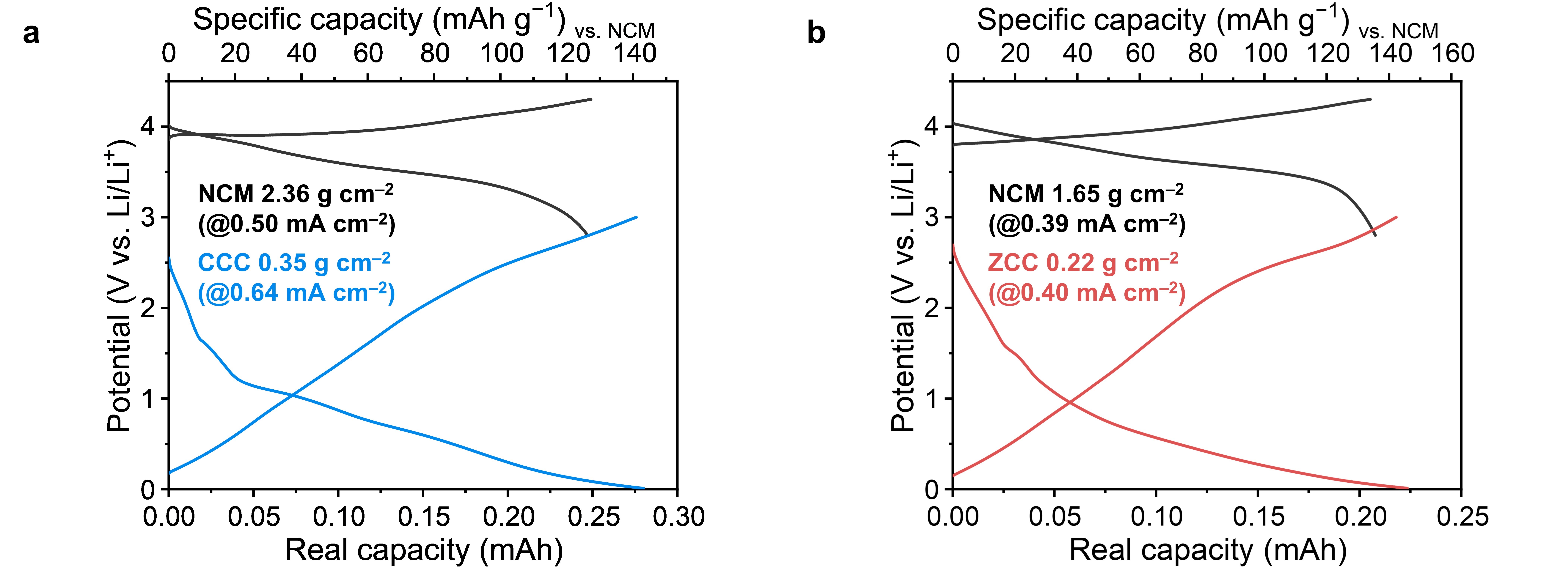


**Figure S7.** Galvanostatic charge-discharge profiles of NCM (black curve) at 0.2 A g^–1^ and MCC anodes in lithium half-cells at 2.0 A g^–1^. a) CCC and b) ZCC anodes.





**Figure S8.** a) Schematic illustration of MCC||NCM full lithium-ion batteries in CR2032 coin-type cells. b) Rate performances of MCC||NCM full lithium-ion batteries at different current densities. c) Cycle performances of MCC||NCM full lithium-ion batteries at 0.1 C. d) Voltage profiles of 2nd to 10th cycles at 0.1 C. e) Voltage profiles of 5, 10, 15, 20, 25th cycles in Figure S8b. (x-axis is the specific capacity versus total mass). f) Ragone plot of MCC||NCM full lithium-ion batteries.


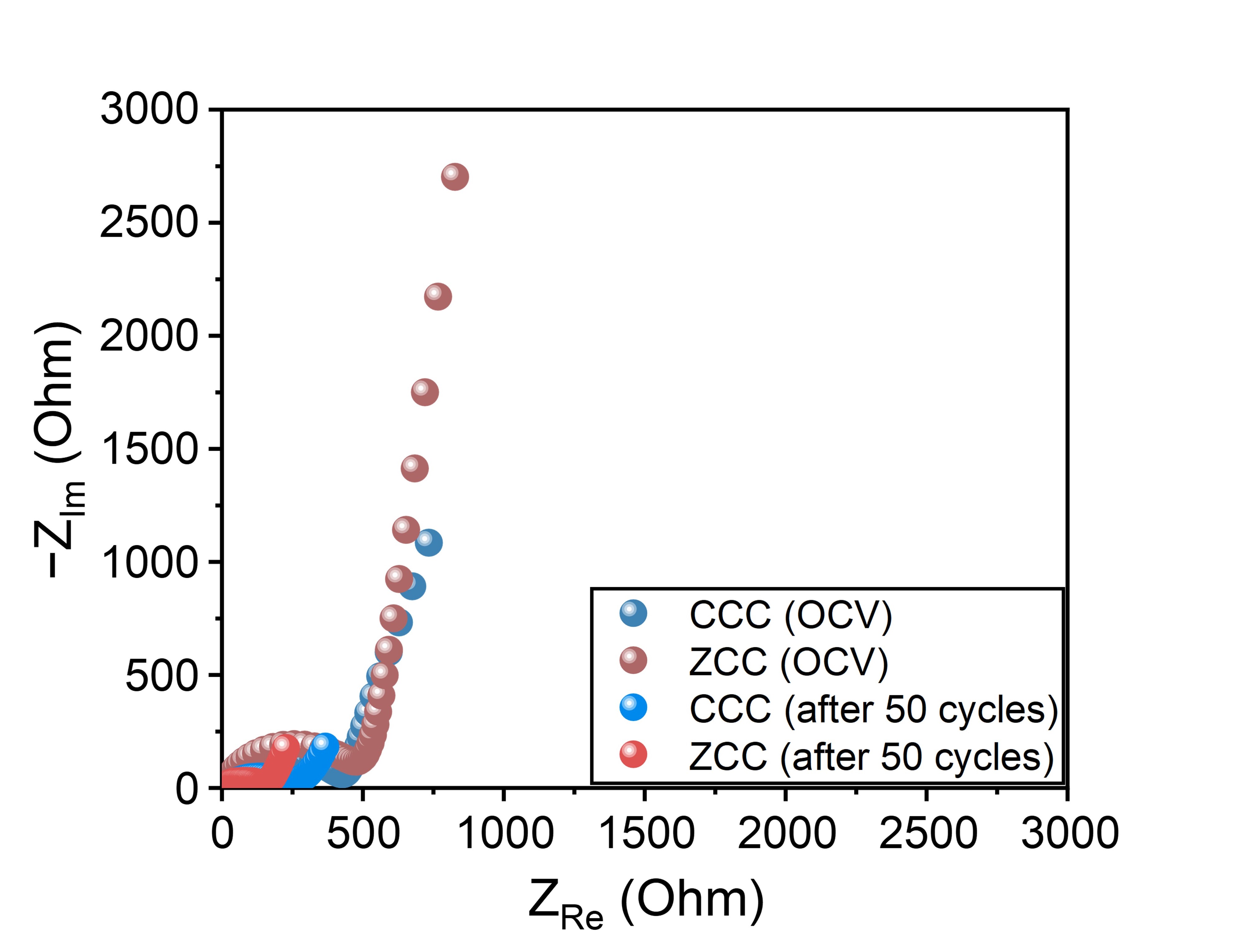


**Figure S9.** Nyquist plots of CCC and ZCC anode at open-circuit voltage state and recharged state after 50 cycles at 0.1 A g^–1^, respectively. (Full spectrum of Figure 4a)


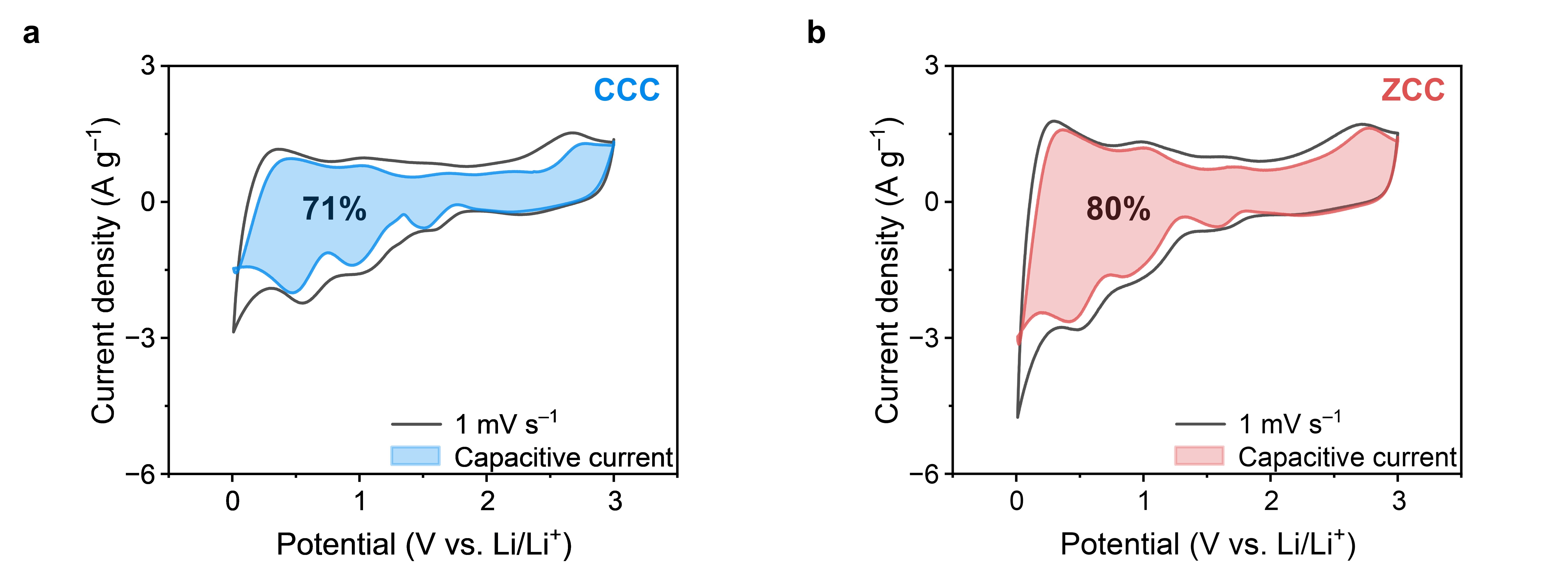


**Figure S10.** Cyclic voltammograms of a) CCC and b) ZCC anodes at 1.0 mV s^–1^ and the visualized capacitive current density.


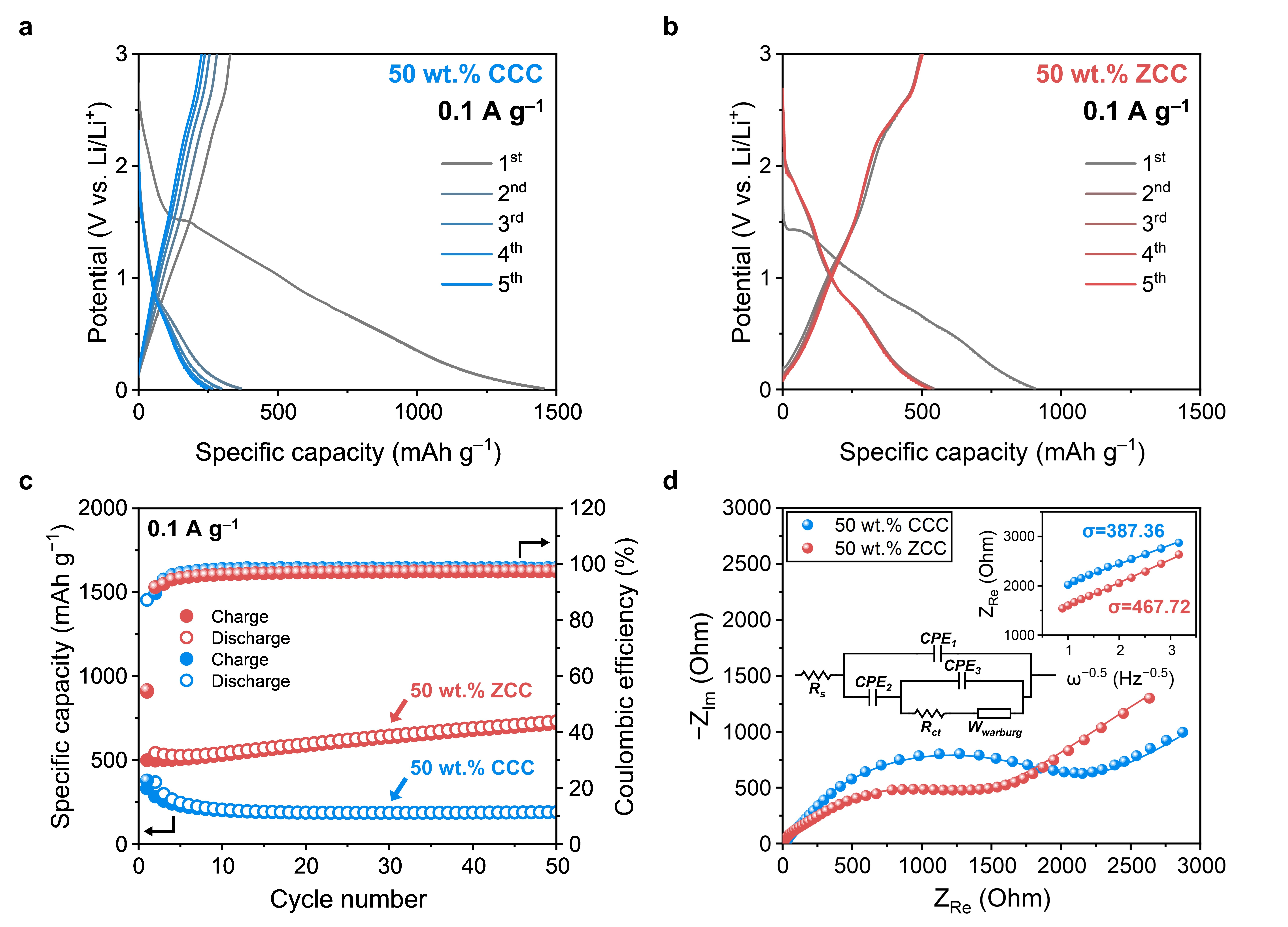


**Figure S11.** Initial 5 galvanostatic charge and discharge profiles of 50 wt.% MCC anodes at 0.1 A g^–1^; a) CCC, b) ZCC. c) Cycle performances of 50 wt.% MCC anodes at 0.1 A g^–1^ over cycle number.

As shown in Figure S11a – S11c, the 50 wt.% MCC anodes (~ 0.6 mg cm^–2^) showed poor reversible capacities at 0.1 A g^–1^, compared to those of 30 wt.% MCC anodes in Figure 2 and 3 of the manuscript. Details are as follows (cycle number, charge capacity (mAh g^–1^), discharge capacity (mAh g^–1^), coulombic efficiency (%)): CCC (1st, 330, 1454, 22.7) (2nd, 281, 368, 89.6) (50th, 187, 189, 98.7); ZCC (1st, 498, 906, 55.0) (2nd, 495, 542, 91.8) (50th, 716, 731, 97.5). Figure S11d displays the Nyquist plots of 50 wt.% CCC and ZCC anodes. And the inset graph shows their linear relationships between the real part of impedance (Z_Re_) and the inverse of the square root of angular frequency (ω) in the low frequency region. Herein, the charge transfer resistance (R_ct_) value of 50 wt.% CCC and ZCC were 1771 and 1327 Ω, and their Li^+^ ion diffusion coefficients (D_Li_) were 3.83 × 10^–13^ and 2.63 × 10^–13^ cm^2^s^–1^, respectively. These deteriorations in cycle performances are caused by their much lower electronic and ionic conductivities than those of 30 wt.% MCC anodes.


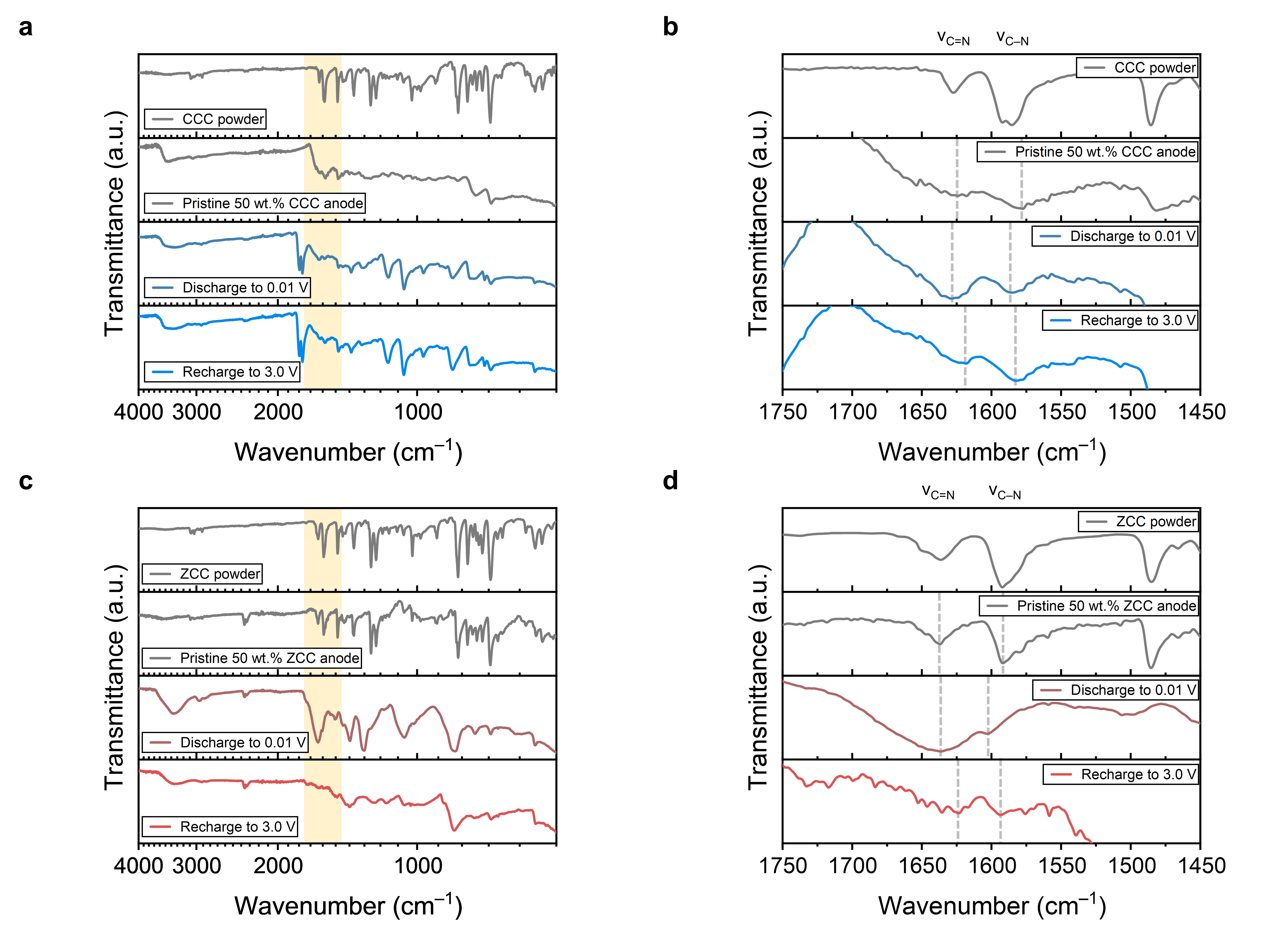


**Figure S12.** Ex-situ ATR FT-IR analysis of a) 50 wt.% CCC and c) 50 wt.% ZCC anodes at different charge levels. The expanded view from 1450 to 1750 cm^–1^; b) 50 wt.% CCC and d) 50 wt.% ZCC anodes.


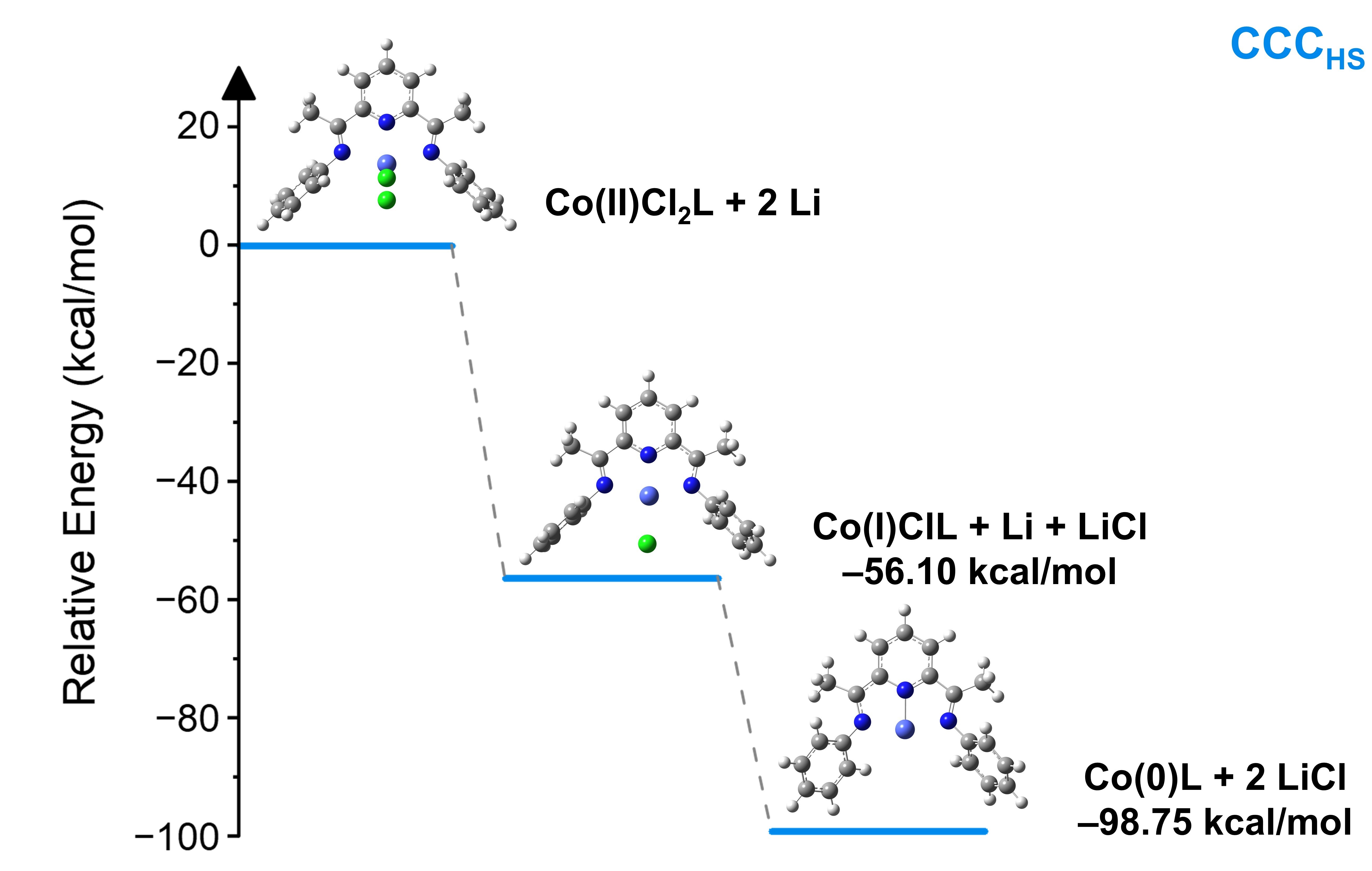


**Figure S13.** DFT-optimized structures and energy differences between each reduced state of CCC_HS_.


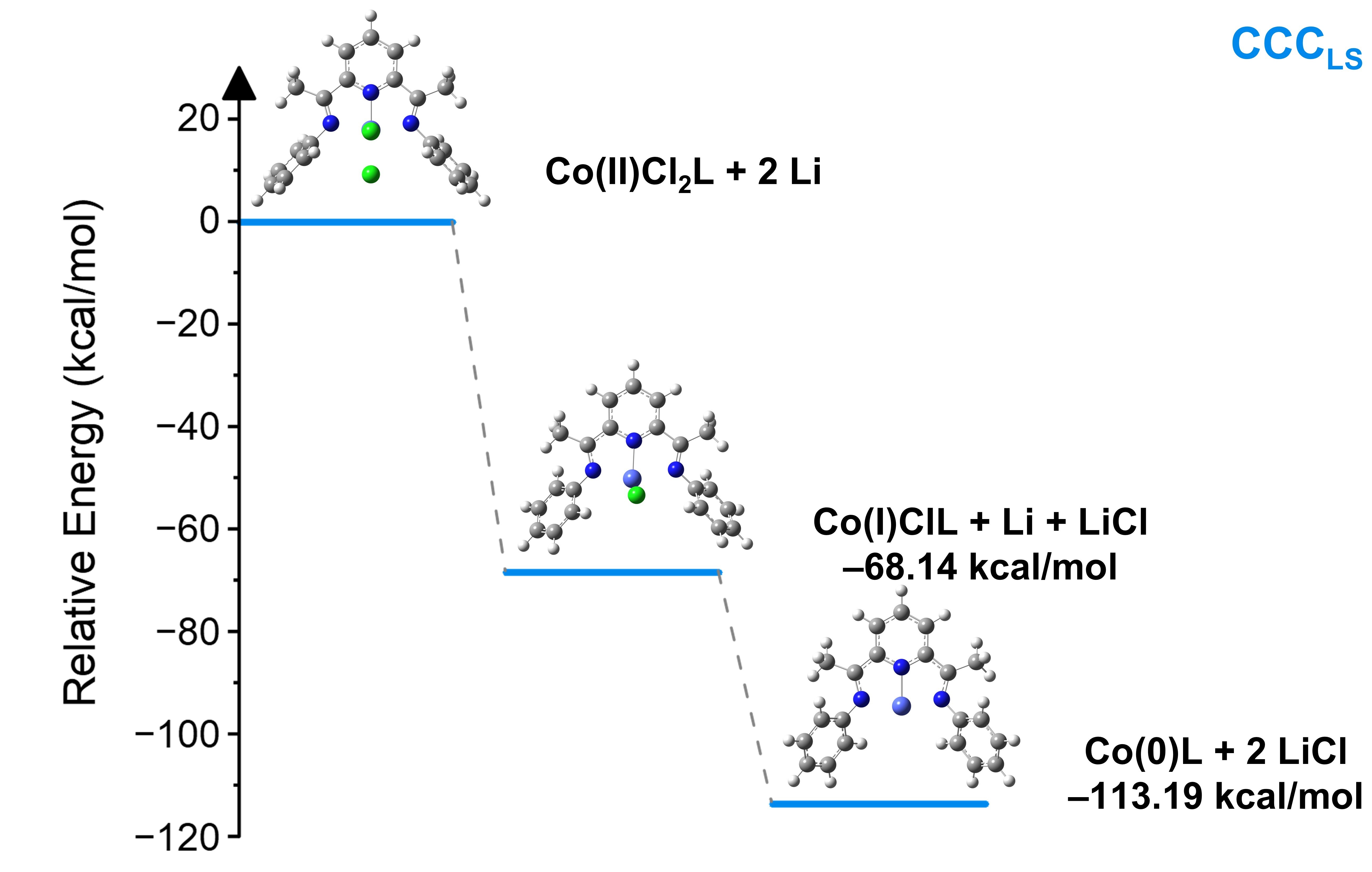


**Figure S14.** DFT-optimized structures and energy differences between each reduced state of CCC_LS_.


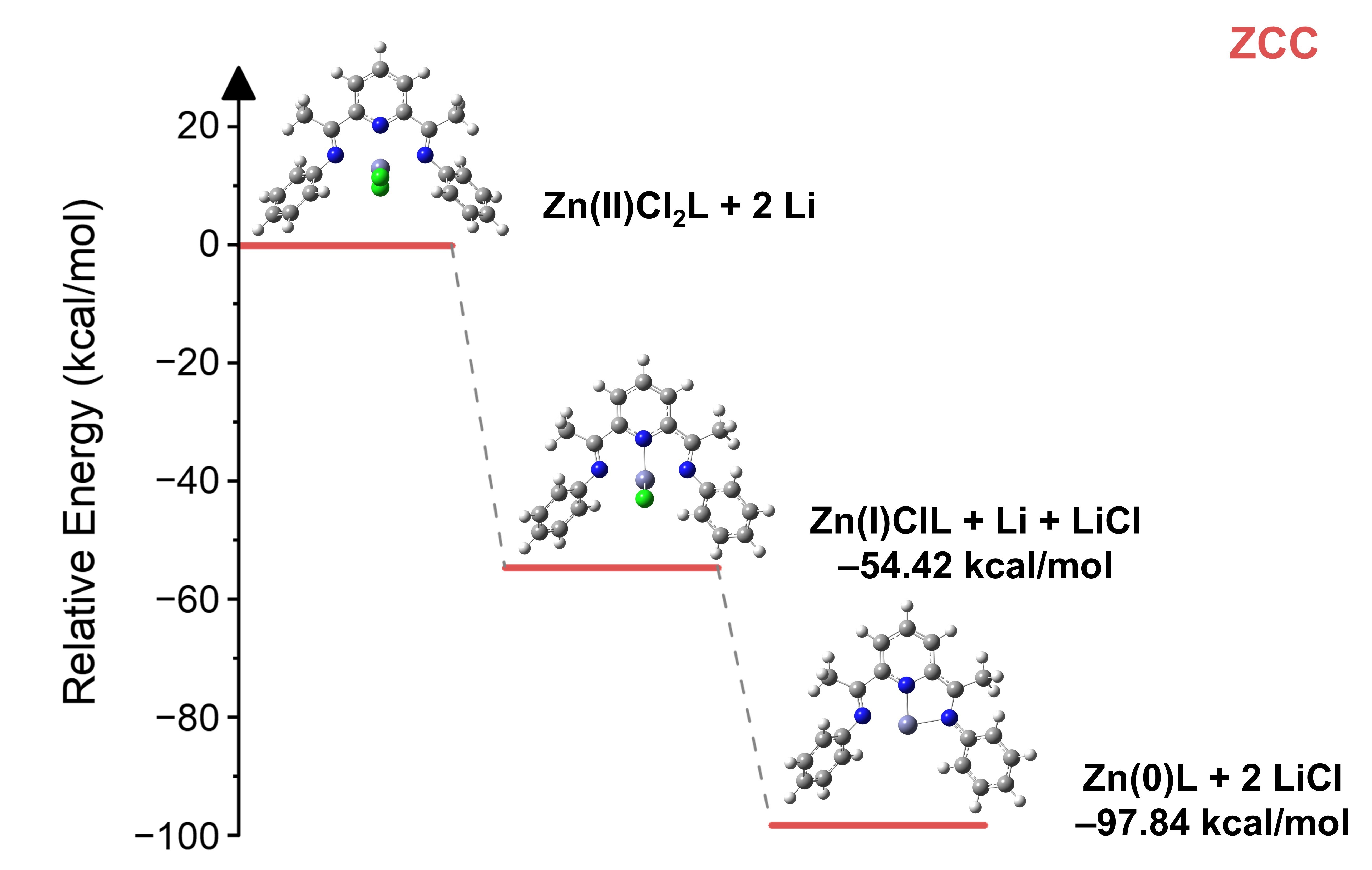


**Figure S15.** DFT-optimized structures and energy differences between each reduced state of ZCC.

**Table S1.** Comparison of cycle performances of MCC anodes and related materials.

| Materials | Capacity  (mAh g^–1^) | Current density  (A g^–1^) | Cycle number | Active material loading (%) | Active material loading  (mg cm^–2^) | Reference |
| --- | --- | --- | --- | --- | --- | --- |
| CoCl_2_ | ~ 400 | *0.2 C | 50 | 65 | ** | ^[1]^ |
| CoCl_2_@CHCB | 920 | 0.2 | 120 | 80 | ~ 1 | ^[2]^ |
|  | 429 | 1 | 200 |  |  |  |
|  | 405 | 2 | 1500 |  |  |  |
| CCH@CM | 909.3 | 0.5 | 200 | 70 | 1.46–1.91 | ^[3]^ |
|  | 769.8 | 1 | 200 |  |  |  |
|  | 540.1 | 2 | 200 |  |  |  |
| CuCl_2_/C | 466 | 0.1 | 20 | 80 | ** | ^[4]^ |
| AgCl/C | 145 | 0.02 | 15 | 80 | ** | ^[4]^ |
|  | 56 | 0.5 | 10 |  |  |  |
| PPy-Fe-O | 820 | 0.1  mA cm^–2^ | 120 | 80 | ** | ^[5]^ |
| PTh–Fe–O | 650 | 0.1  mA cm^–2^ | 300 | 80 | ** | ^[6]^ |
| Zn-LMOF | 623 | 0.05 | 100 | 60 | ** | ^[7]^ |
| CCC | 1630 | 0.1 | 70 | 30 | 0.35 | This work |
|  | 559 | 2.0 | 250 |  |  |  |
| ZCC | 2381 | 0.1 | 100 | 30 | 0.22 |  |
|  | 1720 | 2.0 | 200 |  |  |  |

*There is no description for 1 C.

**No recorded.

References

[1] J. Liu, W. Cui, C. Wang, Y. Xia, *Electrochem commun* **2011**, *13*, 269.

[2] L. Jiang, F. Liang, Z. Zhang, D. Wu, J. Chai, T. Luo, N. Han, W. Zhang, Y. Rui, B. Tang, *Chemical Engineering Journal* **2022**, *433*, 133568.

[3] Y. X. Chen, Y. F. Yuan, Z. J. Yao, M. Zhu, P. F. Du, S. Y. Guo, *Electrochim Acta* **2022**, *430*, 141092.

[4] T. Li, Z. X. Chen, Y. L. Cao, X. P. Ai, H. X. Yang, *Electrochim Acta* **2012**, *68*, 202.

[5] Y. Mao, Q. Kong, B. Guo, X. Fang, X. Guo, L. Shen, M. Armand, Z. Wang, L. Chen, *Energy Environ Sci* **2011**, *4*, 3442.

[6] Y. Mao, Q. Kong, L. Shen, Z. Wang, L. Chen, *J Power Sources* **2014**, *248*, 343.

[7] C. Shi, Y. Gao, L. Liu, Y. Song, X. Wang, H.-J. Liu, Q. Liu, *Journal of Nanoparticle Research* **2016**, *18*, 371.

**Table S2.** Spin equilibrium constant of CCC in each oxidation state.

| K_SC,2+_ | 3.9 ×10^6^ |
| --- | --- |
| K_SC,1+_ | 5.7 ×10^–3^ |
| K_SC,0_ | 1.0 ×10^–4^ |
